# Supplementary material for: Assessment of root phenotypes in mungbean mini-core collection (MMC) from the World Vegetable Center (AVRDC) Taiwan
Source: PLoS One. 2021 Mar 4;16(3):e0247810. doi: 10.1371/journal.pone.0247810 (PMC7932546; doi:10.1371/journal.pone.0247810)
Supplement: S1 Table — (DOCX) [file pone.0247810.s001.docx]

S1 Table. Passport data of AVRDC Mungbean mini core collection

| **S.No.** | **Name of entry** | **Country** | **AVRDC ID** | **Pedigree** |
| --- | --- | --- | --- | --- |
| 1 | IC616240 | Iraq | VI003907 AG | PI 305413 (GREEN) |
| 2 | EC862629 | Thailand | VI002611 AG | (WATT & FINKNER '73) |
| 3 | IC616238 | India | VI003893 AG | M0267 (GREEN) |
| 4 | IC616252 | India | VI004006 A-GM | LM324 (MOTTLE) |
| 5 | IC616275 | India | VI004915 BG | - |
| 6 | IC616273 | India | VI004871 BG | - |
| 7 | IC616274 | India | VI004877 AG | - |
| 8 | IC616110 | India | VI000735 BG | 1791 |
| 9 | IC616157 | India | VI002173 AG | - |
| 10 | EC862600 | Philippines | VI001221 AG | PARC |
| 11 | IC616181 | India | VI003187 BG | - |
| 12 | IC616190 | India | VI003255 AG | 32/2D |
| 13 | IC616216 | India | VI003577 AG | - |
| 14 | EC862611 | United States of America | VI002063 BG | - |
| 15 | IC616136 | India | VI001548 AG | MS 9784 |
| 16 | IC616148 | India | VI001698 BG | G-20 |
| 17 | EC862647 | Afghanistan | VI004145 B-BLM | NEMB- 38 (GREEN) |
| 18 | EC862588 | Pakistan | VI000317 BG | - |
| 19 | IC616134 | India | VI001535 BG | MS 9552 |
| 20 | EC862672 | Pakistan | VI004968 AG | S/6 |
| 21 | IC616123 | India | VI001268 BG | - |
| 22 | EC862602 | Philippines | VI001339 AG | - |
| 23 | IC616135 | India | VI001539 AG | MS 9719 |
| 24 | EC862631 | Thailand | VI002647 AG | (WATT & FINKNER '73) |
| 25 | EC862645 | Australia | VI004024 AG | OSU M- 79 (MOTTLE) |
| 26 | IC616170 | India | VI003057 BG | - |
| 27 | EC862650 | Afghanistan | VI004297 AG | - |
| 28 | EC862595 | Australia | VI001124 AG | CQ16179 |
| 29 | IC616169 | India | VI003035 AG | - |
| 30 | IC616151 | India | VI001743 BG | - |
| 31 | EC15209 | Pakistan | VI001509 AG | EC15209 |
| 32 | IC616204 | India | VI003470 Bg | - |
| 33 | IC616174 | India | VI003083 BG | EB-3 |
| 34 | IC616218 | India | VI003642 AG | - |
| 35 | EC862610 | Korea, Republic of | VI001993 BG | KYUNG-KI JAERAE # 14 |
| 36 | IC616222 | India | VI003678 BG | - |
| 37 | IC616160 | India | VI002176 BG | - |
| 38 | IC616261 | India | VI004244 B-BR | - |
| 39 | EC862591 | Thailand | VI000461 BG | - |
| 40 | EC862609 | Korea, Republic of | VI001974 BG | CHUNG-BUK JAERAE # 24 |
| 41 | EC862634 | Iran | VI002802 A-BR | - |
| 42 | IC616172 | India | VI003068 A-BR | 3 |
| 43 | EC862637 | Iran | VI002872 BG | - |
| 44 | IC616128 | India | VI001403 BR | NP-40 |
| 45 | IC616194 | India | VI003337 BG | - |
| 46 | IC616197 | India | VI003382 BG | - |
| 47 | IC616159 | India | VI002176 AG | - |
| 48 | IC616140 | India | VI001579 BG | C25 |
| 49 | IC616158 | India | VI002173 BG | - |
| 50 | IC616199 | India | VI003413 BG | - |
| 51 | IC616143 | India | VI001651 BG | D-7-55 |
| 52 | EC862667 | Pakistan | VI004954 BG | 71-47 |
| 53 | IC616113 | India | VI000764 AG | - |
| 54 | IC616198 | India | VI003407 AG | - |
| 55 | IC616227 | India | VI003734 B-BR | - |
| 56 | IC616119 | India | VI000938 AG | 12-2/19 (4465X4474) |
| 57 | EC862612 | Thailand | VI002195 AG | MG 55-3 |
| 58 | EC862641 | Unknown | VI003019 BG | - |
| 59 | IC616208 | India | VI003514 BG | - |
| 60 | EC862584 | Thailand | VI000020 AY | CM-16 |
| 61 | IC616235 | India | VI003795 AG | - |
| 62 | IC616242 | India | VI003925 B-BLM | LM055 (BROWN) |
| 63 | IC616258 | India | VI004096 AG | LM203 (GREEN) |
| 64 | IC616256 | India | VI004048 A-GM | M0157 (MOTTLE) |
| 65 | IC616278 | India | VI005066 A-GM | D-48 |
| 66 | EC862628 | Australia | VI002587 AG | CPI 10594 |
| 67 | IC616231 | India | VI003755 BG | - |
| 68 | IC616105 | India | VI000554 AG | NP-21 |
| 69 | IC616267 | India | VI004810 BG | - |
| 70 | EC862658 | Iran | VI004691 AG | - |
| 71 | EC862673 | Pakistan | VI004969 AG | M.M./10 |
| 72 | IC616248 | India | VI003954 BG | LM162 (BROWN) |
| 73 | IC616139 | India | VI001576 BG | 38-1/1 |
| 74 | EC862653 | Iran | VI004423 AG | - |
| 75 | IC616250 | India | VI003958 B-BLM | LM186 (MOTTLE) |
| 76 | EC15131 | India | VI000175 BY | EC15131 |
| 77 | IC616186 | India | VI003242 AG | - |
| 78 | IC616146 | India | VI001678 BG | SELECTION NO18 |
| 79 | EC862659 | Iran | VI004694 BG | - |
| 80 | EC862662 | Pakistan | VI004931 AG | PAK10 |
| 81 | EC862651 | Afghanistan | VI004302 AG | - |
| 82 | IC616191 | India | VI003276 BG | - |
| 83 | EC862665 | Pakistan | VI004937 AG | PAK25 |
| 84 | IC616263 | India | VI004347 B-BLM | -- |
| 85 | EC862638 | Iran | VI002877 BG | - |
| 86 | EC15216 | India | VI001514 AG | EC15216 |
| 87 | EC15125 | Afghanistan | VI000170 B-BR | EC15125 |
| 88 | IC616156 | India | VI002051 BG | - |
| 89 | EC862669 | Pakistan | VI004957 AG | 71-61 |
| 90 | IC616179 | India | VI003181 B-GM | - |
| 91 | IC616235 | India | VI003801 BG | - |
| 92 | IC616176 | India | VI003135 B-BL | 11 MOONG |
| 93 | EC862621 | Korea, Republic of | VI002456 AG | IRI |
| 94 | IC616138 | India | VI001562 AG | P396 |
| 95 | IC616214 | India | VI003560 BG | - |
| 96 | EC862642 | Afghanistan | VI003882 A-BLM | M0231 (BLACK) |
| 97 | EC862619 | Thailand | VI002432 AG | HK-19 (MKT HP 1975) |
| 98 | IC616161 | India | VI002190 BG | - |
| 99 | IC616195 | India | VI003364 AG | - |
| 100 | EC862671 | Pakistan | VI004965 BG | S/425 |
| 101 | IC616196 | India | VI003379 BG | - |
| 102 | EC862592 | Pakistan | VI000470 AG | - |
| 103 | EC862586 | United States of America | VI000212 A-BLM | - |
| 104 | EC862633 | Iran | VI002739 AG | - |
| 105 | IC616129 | India | VI001408 BG | 1-3-126 |
| 106 | EC862597 | Australia | VI001162 AG | CQ29972 |
| 107 | EC862623 | Pakistan | VI002487 AG | 71-27 |
| 108 | IC616154 | India | VI002009 BG | 24-2 |
| 109 | IC616230 | India | VI003744 AG | - |
| 110 | IC616202 | Unknown | VI003456 AG | - |
| 111 | EC16273 | Unknown | VI001612 AG | EC16273 |
| 112 | IC616162 | India | VI002532 AG | BOLANGIR LOCAL |
| 113 | IC616177 | India | VI003159 AG | - |
| 114 | IC616167 | India | VI002999 AG | - |
| 115 | EC862617 | Afghanistan | VI002284 BG | 443-M |
| 116 | IC616200 | India | VI003440 AG | - |
| 117 | EC862649 | Turkey | VI004243 B-BR | - |
| 118 | IC616150 | India | VI001733 BG | - |
| 119 | EC862630 | Thailand | VI002646 AG | (WATT & FINKNER '73) |
| 120 | EC862654 | Iran | VI004432 B-BR | - |
| 121 | EC862615 | Afghanistan | VI002239 AG | 177-M |
| 122 | EC15198 | India | VI000253 AG | EC15198 |
| 123 | IC616125 | India | VI001284 AG | - |
| 124 | IC616224 | India | VI003720 BG | - |
| 125 | IC616212 | India | VI003548 AG | - |
| 126 | IC616182 | India | VI003212 B-BLM | - |
| 127 | IC616122 | India | VI001023 BG | T-44 |
| 128 | IC616116 | India | VI000815 BG | 44-4/1 (T44) (T1XT49) |
| 129 | IC616145 | India | VI001654 BG | D-45-6 |
| 130 | EC862624 | Thailand | VI002523 AG | - |
| 131 | IC616213 | India | VI003554 AG | - |
| 132 | EC862607 | France | VI001820 BG | BF |
| 133 | IC616117 | India | VI000818 BG | 9/11 (T1X48-6) |
| 134 | EC862635 | Iran | VI002859 BG | - |
| 135 | EC862601 | Philippines | VI001244 AG | - |
| 136 | IC616142 | India | VI001628 AG | M-34 |
| 137 | IC616118 | India | VI000852 AG | OB 76-1/2(T1X4425-2) |
| 138 | IC616187 | India | VI003251 A-BL | - |
| 139 | IC616163 | India | VI002926 AG | - |
| 140 | EC15024 | Brazil | VI000616 BG | EC15024 |
| 141 | IC616232 | India | VI003760 BG | - |
| 142 | EC15184 | Afghanistan | VI000238 AG | EC15184 |
| 143 | IC616188 | India | VI003251 A-BLM | - |
| 144 | IC616277 | India | VI005022 BG | 2787 |
| 145 | IC616241 | India | VI003914 AG | LM034 (GREEN) |
| 146 | IC616205 | India | VI003480 BG | - |
| 147 | EC862596 | Australia | VI001126 BG | CQ17415 |
| 148 | EC862655 | Iran | VI004480 AG | - |
| 149 | EC862599 | Philippines | VI001211 AG | - |
| 150 | IC616255-a | India | VI004045 A-GM | LM046 (MOTTLE) |
| 151 | IC616173 | India | VI003070 AG | MU2. |
| 152 | EC862676 | Unknown | VI005041 AG | - |
| 153 | EC862614 | Philippines | VI002206 AG | PHLV#16 (SEL) |
| 154 | EC862644 | Afghanistan | VI003944 B-BR | M0688 (BROWN) |
| 155 | IC616201 | India | VI003455 AG | - |
| 156 | IC616168 | India | VI003034 BG | - |
| 157 | EC862660 | Iran | VI004710 AG | - |
| 158 | IC616257 | India | VI004069 BG | LM124 (GREEN) |
| 159 | IC616260 | India | VI004138 BG | S-9 (LARGE SEED) |
| 160 | IC616269 | India | VI004822 BG | - |
| 161 | EC862643 | Afghanistan | VI003942 AG | M0688 (GREEN) |
| 162 | IC616124 | India | VI001282 AG | - |
| 163 | IC616171 | India | VI003062 BG | - |
| 164 | IC616141 | India | VI001605 BG | 6311 |
| 165 | IC616178 | India | VI003172 BG | YELLOW KHANDALA |
| 166 | IC616219 | India | VI003648 BG | - |
| 167 | EC862613 | Korea, Republic of | VI002197 BG | JAERAE # 4 |
| 168 | IC616251 | India | VI003959 BG | LM194 (GREEN) |
| 169 | EC862664 | Pakistan | VI004934 AG | PAK18 |
| 170 | IC616147 | India | VI001692 AG | P-2-1 |
| 171 | IC616144 | India | VI001652 BG | D-2-15 |
| 172 | EC15181 | Iran | VI001490 AG | EC15181 |
| 173 | EC15158 | Afghanistan | VI000203 B-BR | EC15158 |
| 174 | EC15026 | India | VI000618 AG | EC15026 |
| 175 | EC862668 | Pakistan | VI004956 AG | 71-59 |
| 176 | IC616155 | India | VI002012 BG | T-44 |
| 177 | EC862640 | Unknown | VI003019 A-BLM | - |
| 178 | IC616262 | India | VI004312 AG | - |
| 179 | EC862652 | Afghanistan | VI004307 AG | - |
| 180 | IC616183 | India | VI003220 AG | - |
| 181 | IC616207 | India | VI003493 BG | - |
| 182 | EC862661 | Iran | VI004734 AG | - |
| 183 | IC616112 | India | VI000749 AG | - |
| 184 | EC862622 | Philippines | VI002469 AG | (CES 28 X ML-18) |
| 185 | IC616153 | India | VI001762 A-GM | - |
| 186 | IC616115 | India | VI000805 BG | 24-3/4A (T.1XT49) |
| 187 | IC616209 | India | VI003517 BG | - |
| 188 | EC862590 | Philippines | VI000380 AG | EG-MD-6D |
| 189 | IC616211 | India | VI003534 BG | - |
| 190 | IC616131 | India | VI001412 AG | 1-61-71 |
| 191 | IC616229 | India | VI003734 B-DG | - |
| 192 | IC616223 | India | VI003685 AG | - |
| 193 | IC616114 | India | VI000766 BG | - |
| 194 | IC616127 | India | VI001400 AG | NP-37 |
| 195 | IC616185 | India | VI003235 AG | II-45-89 |
| 196 | IC616268 | India | VI004811 BG | - |
| 197 | EC862646 | Unknown | VI004129 A-BLM | MIDDLE EAST (MOTTLE) |
| 198 | IC616152 | India | VI001756 BG | - |
| 199 | EC862585 | Afghanistan | VI000164 BG | - |
| 200 | IC616271 | India | VI004842 AG | - |
| 201 | EC862594 | Australia | VI001096 AG | Q10590 |
| 202 | IC616247 | India | VI003951 AG | LM154 (MOTTLE) |
| 203 | IC616099 | India | VI000099 AG |  |
| 204 | IC616245 | India | VI003947 B-BR | LM153 (GREEN) |
| 205 | EC15137 | India | VI001471 AG | EC15137 |
| 206 | IC616100 | India | VI000105 BG | - |
| 207 | IC616237 | India | VI003886 BY | M0234 (MOTTLE) |
| 208 | EC15046 | India | VI001448 A-BLM | EC15046 |
| 209 | IC616259 | India | VI004096 BG | LM203 (GREEN) |
| 210 | IC616109 | India | VI000732 AG | N.A.5 |
| 211 | EC862670 | Pakistan | VI004958 BG | 71-72 |
| 212 | EC862603 | Pakistan | VI001406 BG | MOONG 9 |
| 213 | EC862625 | Thailand | VI002529 B-BL | (MARKET, JMP 1972) |
| 214 | IC616239 | India | VI003894 B-BLM | M0267 (BROWN) |
| 215 | EC15179 | Iran | VI000232 AG | EC15179 |
| 216 | IC616265 | India | VI004743 AG | - |
| 217 | EC862663 | Pakistan | VI004933 AG | PAK14 |
| 218 | IC616189 | India | VI003252 BG | - |
| 219 | IC616111 | India | VI000736 AG | M.S.9719 |
| 220 | IC616107 | India | VI000578 AG | 1/28/15 |
| 221 | IC616223 | India | VI003699 B-BG | - |
| 222 | IC616132 | India | VI001419 BG | 1-115-75 |
| 223 | EC15168 | India | VI001482 BG | EC15168 |
| 224 | IC616121 | India | VI000953 AG | PS-7 |
| 225 | EC15020 | United States of America | VI001435 AG | EC15020 |
| 226 | IC616193 | India | VI003332 AG | - |
| 227 | EC862620 | Korea, Republic of | VI002437 BG | BUSAN# 2 |
| 228 | EC862616 | Afghanistan | VI002274 B-BL | 372-M |
| 229 | EC862627 | Nigeria | VI002569 BG | - |
| 230 | IC616264 | India | VI004351 AG | - |
| 231 | IC616226 | India | VI003733 BG | - |
| 232 | EC862677 | Kenya | VI014178 BG | WS80-288 |
| 233 | EC862632 | Thailand | VI002672 AG | (WATT & FINKNER '73) |
| 234 | IC616215 | India | VI003563 A-BR | - |
| 235 | IC616192 | India | VI003329 AG | - |
| 236 | IC616149 | India | VI001728 AG | - |
| 237 | IC616175 | India | VI003114 AG | I-105-30 |
| 238 | EC862636 | Iran | VI002860 AG | - |
| 239 | IC616221 | India | VI003664 AG | - |
| 240 | IC616164 | India | VI002934 AG | - |
| 241 | IC616166 | India | VI002993 BG | - |
| 242 | IC616244 | India | VI003929 A-BL | M0663 (BROWN) |
| 243 | IC616130 | India | VI001411 AG | 1-43-77 |
| 244 | EC862648 | Netherlands | VI004184 AG | NPE 1683 |
| 245 | IC616120 | India | VI000942 AG | - |
| 246 | EC862604 | United States of America | VI001557 BG | M-320 |
| 247 | EC862656 | Iran | VI004639 AG | - |
| 248 | IC616126 | India | VI001385 AG | NP-17-2 |
| 249 | IC616254 | India | VI004044 BG | LM046 (GREEN) |
| 250 | EC862618 | Thailand | VI002402 BG | TH5 (MKT HP 1975) |
| 251 | EC15171 | United States of America | VI000680 AG | EC15171 |
| 252 | IC616106 | India | VI000559 AG | NP-28 |
| 253 | EC862657 | Iran | VI004666 AG | - |
| 254 | IC616203 | India | VI003465 BG | 54 |
| 255 | IC616246 | India | VI003948 B-BR | LM153 (BROWN) |
| 256 | IC616276 | India | VI004973 B-BLM | HYB.4-3 |
| 257 | IC616253 | India | VI004010 AG | M0967 (GREEN) |
| 258 | IC616266 | India | VI004789 BG | - |
| 259 | EC862589 | Pakistan | VI000319 AG | - |
| 260 | IC616234 | India | VI003785 BG | 54 |
| 261 | EC862675 | Mexico | VI005030 BY | NI49 |
| 262 | IC616103 | India | VI000542 BY | NP-11 |
| 263 | IC616225 | India | VI003725 BG | - |
| 264 | IC616270 | India | VI004838 AG | - |
| 265 | IC616102 | India | VI000537 BG | NP-5-2 |
| 266 | EC862598 | Philippines | VI001191 BG | - |
| 267 | IC616243 | India | VI003927 AG | LM057 (GREEN) |
| 268 | IC616133 | India | VI001533 BG | MS 9702/2 |
| 269 | IC616104 | India | VI000551 AG | NP-18 |
| 270 | IC616236 | India | VI003886 B-BR | M0234 (MOTTLE) |
| 271 | IC616217 | India | VI003602 AG | - |
| 272 | EC862674 | Taiwan | VI005024 B-BL | LOCAL CUI |
| 273 | IC616180 | India | VI003183 AG | - |
| 274 | EC862606 | Pakistan | VI001806 BG | - |
| 275 | IC616206 | India | VI003490 AG | - |
| 276 | EC862666 | Pakistan | VI004942 BG | PAK35 |
| 277 | IC616184 | India | VI003232 AG | - |
| 278 | EC15144 | Pakistan | VI000188 A-BLM | EC15144 |
| 279 | IC616210 | India | VI003534 AG | - |
| 280 | IC616249 | India | VI003957 AG | LM186 (GREEN) |
| 281 | IC616108 | India | VI000589 B-BR | 1-130-77 |
| 282 | IC616101 | India | VI000532 BG | - |
| 283 | EC862593 | Australia | VI001066 BG | CPI 27760 |
| 284 | EC862605 | Pakistan | VI001806 AG | - |
| 285 | EC15252 | Iran | VI000723 AG | EC15252 |
| 286 | EC15054 | India | VI000625 B-BR | EC15040 |
| 287 | EC862608 | Thailand | VI001859 BG | TH3 |
| 288 | EC15229 | India | VI001520 A-BLM | EC15229 |
| 289 | EC15006 | Philippines | VI000981 BG | EC15006 PARTO |
| 290 | EC862587 | Pakistan | VI000316 AG | - |
| 291 | IC616137 | India | VI001556 BG | M-3 |
| 292 | EC862639 | Iran | VI002894 B-BR | - |
| 293 | IC616272 | India | VI004853 BG | - |
| 294 | IC616165 | India | VI002986 AG | MARU-2 |
| 295 | EC862626 | Turkey | VI002537 AG | - |
| 296 | IC616220 | India | VI003658 BG | - |
