# Supplementary material for: Assessment of root phenotypes in mungbean mini-core collection (MMC) from the World Vegetable Center (AVRDC) Taiwan
Source: PLoS One. 2021 Mar 4;16(3):e0247810. doi: 10.1371/journal.pone.0247810 (PMC7932546; doi:10.1371/journal.pone.0247810)
Supplement: S2 Table — (DOCX) [file pone.0247810.s002.docx]

S2 Table. Mean scores of the AVRDC mungbean mini core collection for fourteen root traits.

| **S.No** | **Name** | **PRL** | **TPA** | **TSA** | **TRL** | **ARD** | **LPV** | **TRV** | **TRT** | **TRF** | **TRC** | **RDW** | **SDW** | **TDW** | **RSR** |
| --- | --- | --- | --- | --- | --- | --- | --- | --- | --- | --- | --- | --- | --- | --- | --- |
| 1 | IC616240 | 19.40 | 4.13 | 4.70 | 10.46 | 0.71 | 10.46 | 0.04 | 9 | 15 | 1 | 14.00 | 16.30 | 30.30 | 0.86 |
| 2 | EC862629 | 70.67 | 8.76 | 8.06 | 38.03 | 0.60 | 38.03 | 0.11 | 26 | 48 | 3 | 48.00 | 52.20 | 100.20 | 0.92 |
| 3 | IC616238 | 12.65 | 3.11 | 4.06 | 8.78 | 0.74 | 8.78 | 0.04 | 9 | 17 | 1 | 8.00 | 14.40 | 22.40 | 0.56 |
| 4 | IC616252 | 46.97 | 5.78 | 8.13 | 33.57 | 0.59 | 33.57 | 0.09 | 18 | 54 | 5 | 32.00 | 45.61 | 77.61 | 0.70 |
| 5 | IC616275 | 35.04 | 5.02 | 6.99 | 17.92 | 0.60 | 17.92 | 0.05 | 19 | 37 | 0 | 19.00 | 33.50 | 52.50 | 0.57 |
| 6 | IC616273 | 27.75 | 4.70 | 5.91 | 8.58 | 0.69 | 8.58 | 0.03 | 12 | 10 | 0 | 18.00 | 27.80 | 45.80 | 0.65 |
| 7 | IC16274 | 13.16 | 3.05 | 4.32 | 7.81 | 0.82 | 7.81 | 0.04 | 15 | 27 | 2 | 9.00 | 11.10 | 20.10 | 0.81 |
| 8 | IC616110 | 25.36 | 4.70 | 5.40 | 16.61 | 0.69 | 16.61 | 0.06 | 12 | 29 | 0 | 17.20 | 28.00 | 45.20 | 0.61 |
| 9 | IC616157 | 25.69 | 4.13 | 6.22 | 10.91 | 0.68 | 10.91 | 0.04 | 12 | 14 | 0 | 9.00 | 11.40 | 20.40 | 0.79 |
| 10 | EC862600 | 42.79 | 4.45 | 9.11 | 20.93 | 0.65 | 20.26 | 0.07 | 22 | 32 | 5 | 17.00 | 23.50 | 40.50 | 0.72 |
| 11 | IC616181 | 19.67 | 3.68 | 4.85 | 11.49 | 0.71 | 10.81 | 0.05 | 21 | 15 | 0 | 35.00 | 45.60 | 80.60 | 0.77 |
| 12 | IC616190 | 33.45 | 4.76 | 6.93 | 24.95 | 0.73 | 24.83 | 0.10 | 25 | 27 | 1 | 9.00 | 11.80 | 20.80 | 0.76 |
| 13 | IC616216 | 14.88 | 2.86 | 5.21 | 4.81 | 1.29 | 4.81 | 0.06 | 2 | 0 | 0 | 77.00 | 93.20 | 170.20 | 0.83 |
| 14 | EC862611 | 22.14 | 3.87 | 5.72 | 15.26 | 0.54 | 15.26 | 0.04 | 21 | 20 | 2 | 5.00 | 5.10 | 10.10 | 0.98 |
| 15 | IC616136 | 55.78 | 6.35 | 8.22 | 18.37 | 0.55 | 17.70 | 0.04 | 22 | 21 | 2 | 64.00 | 86.50 | 150.50 | 0.74 |
| 16 | IC616148 | 47.13 | 4.95 | 8.98 | 17.62 | 0.61 | 16.94 | 0.05 | 18 | 18 | 3 | 48.00 | 32.40 | 80.40 | 1.48 |
| 17 | EC862647 | 24.83 | 4.32 | 5.66 | 19.12 | 0.85 | 19.00 | 0.11 | 26 | 54 | 1 | 18.00 | 32.30 | 50.30 | 0.56 |
| 18 | EC862588 | 35.61 | 4.38 | 8.13 | 19.07 | 0.63 | 19.07 | 0.06 | 25 | 27 | 4 | 8.00 | 2.70 | 10.70 | 2.96 |
| 19 | IC616134 | 10.11 | 2.41 | 4.19 | 4.40 | 1.32 | 4.40 | 0.06 | 2 | 0 | 0 | 73.21 | 97.59 | 170.80 | 0.75 |
| 20 | EC862672 | 20.62 | 3.81 | 4.91 | 8.85 | 0.88 | 8.18 | 0.05 | 10 | 8 | 0 | 62.00 | 68.40 | 130.40 | 0.91 |
| 21 | IC616123 | 39.25 | 3.81 | 9.80 | 15.98 | 0.68 | 15.31 | 0.06 | 25 | 21 | 2 | 9.00 | 11.60 | 20.60 | 0.78 |
| 22 | EC862602 | 25.16 | 3.62 | 6.87 | 14.97 | 0.54 | 14.85 | 0.04 | 15 | 21 | 1 | 8.00 | 2.10 | 10.10 | 3.81 |
| 23 | IC616135 | 17.78 | 2.86 | 6.22 | 7.32 | 0.75 | 7.32 | 0.03 | 5 | 3 | 0 | 25.00 | 35.50 | 60.50 | 0.70 |
| 24 | EC862631 | 29.27 | 3.81 | 7.68 | 16.05 | 0.73 | 16.05 | 0.07 | 20 | 28 | 0 | 59.00 | 61.30 | 120.30 | 0.96 |
| 25 | EC862645 | 40.70 | 5.72 | 6.57 | 40.32 | 0.77 | 39.64 | 0.19 | 41 | 90 | 6 | 36.00 | 44.40 | 80.40 | 0.81 |
| 26 | IC616170 | 34.25 | 3.87 | 8.34 | 11.69 | 0.67 | 11.01 | 0.04 | 15 | 13 | 1 | 4.00 | 6.40 | 10.40 | 0.63 |
| 27 | EC862650 | 39.72 | 4.83 | 8.14 | 25.56 | 0.69 | 25.44 | 0.10 | 24 | 52 | 4 | 8.00 | 12.60 | 20.60 | 0.63 |
| 28 | EC862595 | 36.33 | 4.89 | 7.43 | 14.23 | 0.76 | 14.23 | 0.06 | 21 | 11 | 0 | 35.00 | 45.20 | 80.20 | 0.77 |
| 29 | IC616169 | 19.95 | 3.24 | 6.16 | 16.28 | 0.76 | 16.28 | 0.07 | 27 | 44 | 2 | 19.00 | 11.10 | 30.10 | 1.71 |
| 30 | IC616151 | 36.39 | 5.78 | 5.74 | 33.74 | 0.65 | 33.07 | 0.11 | 22 | 52 | 1 | 4.00 | 6.60 | 10.60 | 0.61 |
| 31 | EC15209 | 45.20 | 5.02 | 8.47 | 17.20 | 0.63 | 16.52 | 0.05 | 26 | 29 | 0 | 9.00 | 11.90 | 20.90 | 0.76 |
| 32 | EC616204 | 49.12 | 5.46 | 8.90 | 21.41 | 0.67 | 21.29 | 0.07 | 28 | 36 | 2 | 36.00 | 44.80 | 80.80 | 0.80 |
| 33 | IC616174 | 35.66 | 4.19 | 8.51 | 20.09 | 0.56 | 20.09 | 0.05 | 24 | 28 | 0 | 16.00 | 24.20 | 40.20 | 0.66 |
| 34 | IC616218 | 15.93 | 3.18 | 5.02 | 16.52 | 0.76 | 16.52 | 0.08 | 21 | 55 | 2 | 12.00 | 18.10 | 30.10 | 0.66 |
| 35 | EC862610 | 23.66 | 4.51 | 4.72 | 12.47 | 0.71 | 11.80 | 0.05 | 18 | 22 | 1 | 41.00 | 49.60 | 90.60 | 0.83 |
| 36 | IC616222 | 12.08 | 3.94 | 2.56 | 3.69 | 1.65 | 3.02 | 0.08 | 10 | 0 | 0 | 29.99 | 70.31 | 100.30 | 0.43 |
| 37 | IC616160 | 38.49 | 5.33 | 7.12 | 19.44 | 0.69 | 19.32 | 0.07 | 32 | 34 | 1 | 4.00 | 6.40 | 10.40 | 0.63 |
| 38 | IC616261 | 14.76 | 3.81 | 3.87 | 4.63 | 1.10 | 4.63 | 0.04 | 4 | 0 | 0 | 42.00 | 48.20 | 90.20 | 0.87 |
| 39 | EC862591 | 19.94 | 3.05 | 6.54 | 6.27 | 0.84 | 6.27 | 0.03 | 10 | 4 | 0 | 26.00 | 34.20 | 60.20 | 0.76 |
| 40 | EC862609 | 8.10 | 2.03 | 3.64 | 3.58 | 0.97 | 2.91 | 0.03 | 3 | 1 | 0 | 42.00 | 48.40 | 90.40 | 0.87 |
| 41 | EC862634 | 2.13 | 1.52 | 1.17 | 1.34 | 1.03 | 0.67 | 0.01 | 4 | 0 | 0 | 39.00 | 61.50 | 100.50 | 0.63 |
| 42 | IC616172 | 7.47 | 3.62 | 1.98 | 3.09 | 1.08 | 2.97 | 0.03 | 2 | 0 | 0 | 33.00 | 57.80 | 90.80 | 0.57 |
| 43 | EC862637 | 12.18 | 3.62 | 3.37 | 4.29 | 1.09 | 4.29 | 0.04 | 8 | 0 | 0 | 28.77 | 51.93 | 80.70 | 0.55 |
| 44 | IC616128 | 13.69 | 2.22 | 6.16 | 12.15 | 0.78 | 12.15 | 0.06 | 14 | 34 | 0 | 41.00 | 69.90 | 110.90 | 0.59 |
| 45 | IC616194 | 4.12 | 2.22 | 1.49 | 2.95 | 1.01 | 2.28 | 0.02 | 4 | 0 | 0 | 35.00 | 65.80 | 100.80 | 0.53 |
| 46 | IC616197 | 11.83 | 3.18 | 3.26 | 5.49 | 0.55 | 4.82 | 0.01 | 14 | 4 | 1 | 8.00 | 12.50 | 20.50 | 0.64 |
| 47 | IC616159 | 23.13 | 5.02 | 4.52 | 6.96 | 0.85 | 6.84 | 0.04 | 6 | 2 | 0 | 52.00 | 98.60 | 150.60 | 0.53 |
| 48 | IC616140 | 20.50 | 3.94 | 5.21 | 12.77 | 0.71 | 12.77 | 0.05 | 20 | 18 | 0 | 21.00 | 39.20 | 60.20 | 0.54 |
| 49 | IC616158 | 10.16 | 4.57 | 2.22 | 4.40 | 0.87 | 4.40 | 0.03 | 4 | 0 | 0 | 42.00 | 68.10 | 110.10 | 0.62 |
| 50 | IC616199 | 4.56 | 1.40 | 3.07 | 2.92 | 0.88 | 2.24 | 0.02 | 5 | 1 | 0 | 36.00 | 44.30 | 80.30 | 0.81 |
| 51 | IC616143 | 16.26 | 4.57 | 3.56 | 4.56 | 0.90 | 4.56 | 0.03 | 2 | 0 | 0 | 12.00 | 18.50 | 30.50 | 0.65 |
| 52 | EC862667 | 27.00 | 4.13 | 6.54 | 19.29 | 0.69 | 19.29 | 0.07 | 21 | 41 | 2 | 8.20 | 12.30 | 20.50 | 0.67 |
| 53 | IC616113 | 23.42 | 3.68 | 5.87 | 15.41 | 0.59 | 14.74 | 0.04 | 19 | 25 | 0 | 31.40 | 49.20 | 80.60 | 0.64 |
| 54 | IC616198 | 7.31 | 2.86 | 2.12 | 2.58 | 1.00 | 1.90 | 0.02 | 2 | 0 | 0 | 36.40 | 63.60 | 100.00 | 0.57 |
| 55 | IC616227 | 34.90 | 5.46 | 6.29 | 18.82 | 0.67 | 18.70 | 0.07 | 22 | 20 | 1 | 32.41 | 37.59 | 70.00 | 0.86 |
| 56 | IC616119 | 14.97 | 4.06 | 3.68 | 13.02 | 0.66 | 13.02 | 0.05 | 12 | 14 | 0 | 31.21 | 38.79 | 70.00 | 0.80 |
| 57 | EC862612 | 9.35 | 3.87 | 2.41 | 3.87 | 1.03 | 3.87 | 0.03 | 4 | 0 | 0 | 64.35 | 95.65 | 160.00 | 0.67 |
| 58 | EC862641 | 5.85 | 2.10 | 2.44 | 3.47 | 1.05 | 2.80 | 0.03 | 4 | 0 | 0 | 33.65 | 66.35 | 100.00 | 0.51 |
| 59 | IC616208 | 67.14 | 7.68 | 8.15 | 38.09 | 0.64 | 37.42 | 0.12 | 40 | 46 | 5 | 9.77 | 20.23 | 30.00 | 0.48 |
| 60 | EC862684 | 5.55 | 2.35 | 2.29 | 1.95 | 1.33 | 1.83 | 0.03 | 2 | 0 | 0 | 64.22 | 95.78 | 160.00 | 0.67 |
| 61 | IC616235 | 13.35 | 3.05 | 4.38 | 3.47 | 1.16 | 3.47 | 0.04 | 4 | 0 | 0 | 4.25 | 5.75 | 10.00 | 0.74 |
| 62 | IC616242 | 16.04 | 3.24 | 4.95 | 13.29 | 0.76 | 13.29 | 0.06 | 22 | 22 | 0 | 4.36 | 5.64 | 10.00 | 0.77 |
| 63 | IC616258 | 22.70 | 4.38 | 4.66 | 16.80 | 0.75 | 16.13 | 0.07 | 18 | 15 | 1 | 4.58 | 5.42 | 10.00 | 0.85 |
| 64 | IC616256 | 26.05 | 4.51 | 5.78 | 10.53 | 0.63 | 10.53 | 0.03 | 15 | 11 | 0 | 9.66 | 20.34 | 30.00 | 0.48 |
| 65 | IC616278 | 33.87 | 3.81 | 8.89 | 14.80 | 0.74 | 14.80 | 0.06 | 25 | 23 | 0 | 24.34 | 55.67 | 80.00 | 0.44 |
| 66 | EC862628 | 14.74 | 3.11 | 4.28 | 10.57 | 0.60 | 9.90 | 0.03 | 17 | 15 | 0 | 4.21 | 5.79 | 10.00 | 0.73 |
| 67 | IC616231 | 32.83 | 4.25 | 7.20 | 12.74 | 0.56 | 12.07 | 0.03 | 17 | 17 | 0 | 26.55 | 53.45 | 80.00 | 0.50 |
| 68 | IC616105 | 16.86 | 3.43 | 4.83 | 12.66 | 0.99 | 12.54 | 0.10 | 9 | 16 | 0 | 15.68 | 34.32 | 50.00 | 0.46 |
| 69 | IC616267 | 7.87 | 2.03 | 3.87 | 3.87 | 1.01 | 3.87 | 0.03 | 8 | 0 | 0 | 8.24 | 11.77 | 20.00 | 0.70 |
| 70 | EC862658 | 9.74 | 2.22 | 4.38 | 3.89 | 0.94 | 3.89 | 0.03 | 6 | 0 | 0 | 9.56 | 16.44 | 26.00 | 0.58 |
| 71 | EC862673 | 15.33 | 3.11 | 4.47 | 4.60 | 0.73 | 3.93 | 0.02 | 6 | 0 | 0 | 14.22 | 20.78 | 35.00 | 0.68 |
| 72 | IC616248 | 6.59 | 2.16 | 2.69 | 3.10 | 1.01 | 2.43 | 0.03 | 4 | 0 | 0 | 38.65 | 81.35 | 120.00 | 0.48 |
| 73 | IC616139 | 13.67 | 3.62 | 3.69 | 5.37 | 0.95 | 5.25 | 0.04 | 4 | 0 | 0 | 35.24 | 64.76 | 100.00 | 0.54 |
| 74 | EC862653 | 5.67 | 2.35 | 2.41 | 2.24 | 1.74 | 2.24 | 0.05 | 2 | 0 | 0 | 24.32 | 55.68 | 80.00 | 0.44 |
| 75 | IC616250 | 6.58 | 3.24 | 2.03 | 1.52 | 1.29 | 1.52 | 0.02 | 2 | 0 | 0 | 15.60 | 34.40 | 50.00 | 0.45 |
| 76 | EC15131 | 21.50 | 3.49 | 5.68 | 5.88 | 0.69 | 5.21 | 0.02 | 4 | 0 | 0 | 4.20 | 5.80 | 10.00 | 0.72 |
| 77 | IC616186 | 20.23 | 3.62 | 5.59 | 6.60 | 0.72 | 6.60 | 0.03 | 8 | 0 | 1 | 55.64 | 84.36 | 140.00 | 0.66 |
| 78 | IC616146 | 12.48 | 2.16 | 5.78 | 4.92 | 0.90 | 4.92 | 0.03 | 6 | 0 | 0 | 4.00 | 6.00 | 10.00 | 0.67 |
| 79 | EC862659 | 4.92 | 2.10 | 1.99 | 2.06 | 1.15 | 1.39 | 0.02 | 2 | 0 | 0 | 5.00 | 5.00 | 10.00 | 1.00 |
| 80 | EC862662 | 2.59 | 1.91 | 1.04 | 0.79 | 1.15 | 0.12 | 0.01 | 2 | 0 | 0 | 4.50 | 85.50 | 90.00 | 0.05 |
| 81 | EC862651 | 3.87 | 2.73 | 1.34 | 1.78 | 1.36 | 1.66 | 0.03 | 4 | 0 | 0 | 35.66 | 84.34 | 120.00 | 0.42 |
| 82 | IC616191 | 4.66 | 2.16 | 2.16 | 2.44 | 1.35 | 2.44 | 0.04 | 2 | 0 | 0 | 41.22 | 78.78 | 120.00 | 0.52 |
| 83 | EC862665 | 12.16 | 2.86 | 4.25 | 4.55 | 0.72 | 4.55 | 0.02 | 4 | 0 | 0 | 21.67 | 58.34 | 80.00 | 0.37 |
| 84 | IC616263 | 37.59 | 4.64 | 7.58 | 13.29 | 0.60 | 12.61 | 0.04 | 25 | 15 | 0 | 24.13 | 95.87 | 120.00 | 0.25 |
| 85 | EC862638 | 18.87 | 3.05 | 5.74 | 6.88 | 0.87 | 6.21 | 0.04 | 4 | 0 | 0 | 8.24 | 11.76 | 20.00 | 0.70 |
| 86 | EC15216 | 15.58 | 3.75 | 4.07 | 5.08 | 0.79 | 4.96 | 0.03 | 7 | 1 | 0 | 4.36 | 5.64 | 10.00 | 0.77 |
| 87 | EC15125 | 38.21 | 5.14 | 7.43 | 21.19 | 0.85 | 21.19 | 0.12 | 31 | 40 | 3 | 33.58 | 66.42 | 100.00 | 0.51 |
| 88 | IC616156 | 30.06 | 4.51 | 6.67 | 14.11 | 0.61 | 14.11 | 0.04 | 14 | 12 | 0 | 10.24 | 19.76 | 30.00 | 0.52 |
| 89 | EC862669 | 39.36 | 4.32 | 8.60 | 23.73 | 0.47 | 23.05 | 0.04 | 26 | 22 | 3 | 21.00 | 29.00 | 50.00 | 0.72 |
| 90 | IC616179 | 10.28 | 3.18 | 3.24 | 5.95 | 0.94 | 5.95 | 0.04 | 4 | 0 | 0 | 24.22 | 35.78 | 60.00 | 0.68 |
| 91 | IC616235 | 22.21 | 3.24 | 6.86 | 10.80 | 0.62 | 10.80 | 0.03 | 11 | 7 | 0 | 36.24 | 53.76 | 90.00 | 0.67 |
| 92 | IC616176 | 5.53 | 1.71 | 2.95 | 3.16 | 0.93 | 2.49 | 0.02 | 2 | 0 | 0 | 4.21 | 5.79 | 10.00 | 0.73 |
| 93 | EC862621 | 25.97 | 3.56 | 6.82 | 16.09 | 0.68 | 15.42 | 0.06 | 20 | 21 | 2 | 25.21 | 44.79 | 70.00 | 0.56 |
| 94 | IC616138 | 12.93 | 2.48 | 5.15 | 8.69 | 0.67 | 8.57 | 0.03 | 18 | 11 | 0 | 14.34 | 35.67 | 50.00 | 0.40 |
| 95 | IC616214 | 58.06 | 6.10 | 9.53 | 34.51 | 0.64 | 34.51 | 0.11 | 36 | 50 | 3 | 15.23 | 34.78 | 50.00 | 0.44 |
| 96 | EC862642 | 26.67 | 4.00 | 6.67 | 13.99 | 0.75 | 13.99 | 0.06 | 37 | 29 | 1 | 24.11 | 35.89 | 60.00 | 0.67 |
| 97 | EC862619 | 37.21 | 3.68 | 9.61 | 15.37 | 0.55 | 14.70 | 0.04 | 38 | 24 | 0 | 46.25 | 93.75 | 140.00 | 0.49 |
| 98 | IC616161 | 12.59 | 2.98 | 3.77 | 5.49 | 0.81 | 4.81 | 0.03 | 8 | 2 | 0 | 15.68 | 24.32 | 40.00 | 0.64 |
| 99 | IC616195 | 4.33 | 1.52 | 2.80 | 1.60 | 1.30 | 1.48 | 0.02 | 2 | 0 | 0 | 35.66 | 84.34 | 120.00 | 0.42 |
| 100 | EC862671 | 9.87 | 3.24 | 3.05 | 3.03 | 1.23 | 3.03 | 0.04 | 4 | 0 | 0 | 41.33 | 88.67 | 130.00 | 0.47 |
| 101 | IC616196 | 33.31 | 4.45 | 7.49 | 21.24 | 0.71 | 21.24 | 0.09 | 34 | 43 | 1 | 9.35 | 20.65 | 30.00 | 0.45 |
| 102 | EC862692 | 66.45 | 5.91 | 10.69 | 19.33 | 0.57 | 18.65 | 0.05 | 28 | 16 | 3 | 33.65 | 66.35 | 100.00 | 0.51 |
| 103 | EC862586 | 45.58 | 5.40 | 8.45 | 15.92 | 0.62 | 15.92 | 0.05 | 20 | 25 | 3 | 24.29 | 45.71 | 70.00 | 0.53 |
| 104 | EC862633 | 9.00 | 2.29 | 3.94 | 3.04 | 0.83 | 3.97 | 0.02 | 4 | 0 | 0 | 4.24 | 5.76 | 10.00 | 0.74 |
| 105 | IC616129 | 8.83 | 1.97 | 4.15 | 6.54 | 0.63 | 4.74 | 0.02 | 2 | 0 | 0 | 4.67 | 6.33 | 11.00 | 0.74 |
| 106 | EC862597 | 59.47 | 8.38 | 6.50 | 35.91 | 0.63 | 35.24 | 0.11 | 31 | 27 | 2 | 35.15 | 54.85 | 90.00 | 0.64 |
| 107 | EC862623 | 17.27 | 2.79 | 6.10 | 7.77 | 0.84 | 7.65 | 0.04 | 10 | 6 | 0 | 36.55 | 53.45 | 90.00 | 0.68 |
| 108 | IC616154 | 1.96 | 1.14 | 1.71 | 1.66 | 0.78 | 1.66 | 0.01 | 2 | 0 | 0 | 4.55 | 5.45 | 10.00 | 0.84 |
| 109 | IC616230 | 10.84 | 2.03 | 5.33 | 5.52 | 0.94 | 5.52 | 0.04 | 8 | 2 | 0 | 55.47 | 84.53 | 140.00 | 0.66 |
| 110 | IC616202 | 39.01 | 4.70 | 7.77 | 23.80 | 0.55 | 23.13 | 0.06 | 36 | 33 | 0 | 8.55 | 16.45 | 25.00 | 0.52 |
| 111 | EC16273 | 8.04 | 3.43 | 1.87 | 3.75 | 1.04 | 3.08 | 0.03 | 6 | 0 | 0 | 54.33 | 95.67 | 150.00 | 0.57 |
| 112 | IC616162 | 28.66 | 3.87 | 7.31 | 12.58 | 0.65 | 12.46 | 0.04 | 30 | 14 | 2 | 35.21 | 55.01 | 90.22 | 0.64 |
| 113 | IC616177 | 33.53 | 4.00 | 8.38 | 13.85 | 0.69 | 13.85 | 0.05 | 29 | 17 | 0 | 26.55 | 57.67 | 84.22 | 0.46 |
| 114 | IC616167 | 49.23 | 4.19 | 11.75 | 24.00 | 0.53 | 24.00 | 0.05 | 41 | 23 | 3 | 24.01 | 40.10 | 64.11 | 0.60 |
| 115 | EC862617 | 54.68 | 6.10 | 8.41 | 27.98 | 0.66 | 27.31 | 0.10 | 46 | 38 | 4 | 99.89 | 177.11 | 277.00 | 0.56 |
| 116 | IC616200 | 23.15 | 3.68 | 6.29 | 13.16 | 0.64 | 13.16 | 0.04 | 15 | 15 | 2 | 88.00 | 139.00 | 227.00 | 0.63 |
| 117 | EC862649 | 21.75 | 4.13 | 5.27 | 19.28 | 0.64 | 19.28 | 0.06 | 26 | 41 | 0 | 15.00 | 35.00 | 50.00 | 0.43 |
| 118 | IC616150 | 17.62 | 3.56 | 4.47 | 15.73 | 0.63 | 15.05 | 0.05 | 18 | 38 | 2 | 80.64 | 139.36 | 220.00 | 0.58 |
| 119 | EC862630 | 5.42 | 2.29 | 1.99 | 3.75 | 0.82 | 3.08 | 0.02 | 6 | 0 | 0 | 24.34 | 35.67 | 60.00 | 0.68 |
| 120 | EC862654 | 32.82 | 5.46 | 5.91 | 11.37 | 0.60 | 11.25 | 0.03 | 20 | 6 | 0 | 78.21 | 152.79 | 231.00 | 0.51 |
| 121 | IC862615 | 39.79 | 4.51 | 8.83 | 24.58 | 0.67 | 24.58 | 0.09 | 34 | 32 | 3 | 99.97 | 161.04 | 261.00 | 0.62 |
| 122 | EC15198 | 29.72 | 4.25 | 6.99 | 18.51 | 0.78 | 18.51 | 0.09 | 28 | 42 | 0 | 68.95 | 141.05 | 210.00 | 0.49 |
| 123 | IC616125 | 7.30 | 2.73 | 2.25 | 4.38 | 0.83 | 3.71 | 0.02 | 4 | 1 | 0 | 35.47 | 54.53 | 90.00 | 0.65 |
| 124 | IC616224 | 27.63 | 5.72 | 4.28 | 27.96 | 0.70 | 27.28 | 0.11 | 21 | 25 | 1 | 75.22 | 133.78 | 209.00 | 0.56 |
| 125 | IC616212 | 8.49 | 2.22 | 3.75 | 3.28 | 1.02 | 3.16 | 0.03 | 2 | 0 | 0 | 38.66 | 91.34 | 130.00 | 0.42 |
| 126 | IC616182 | 33.97 | 4.57 | 7.43 | 12.69 | 0.66 | 12.69 | 0.04 | 13 | 5 | 0 | 25.44 | 38.56 | 64.00 | 0.66 |
| 127 | IC616122 | 21.50 | 3.94 | 5.46 | 8.83 | 0.70 | 8.83 | 0.03 | 7 | 5 | 1 | 35.50 | 49.50 | 85.00 | 0.72 |
| 128 | IC616116 | 8.04 | 1.91 | 3.90 | 3.80 | 1.02 | 3.12 | 0.03 | 3 | 1 | 0 | 36.54 | 83.46 | 120.00 | 0.44 |
| 129 | IC616145 | 45.08 | 5.46 | 8.26 | 19.70 | 0.69 | 19.70 | 0.07 | 29 | 26 | 0 | 24.34 | 55.66 | 80.00 | 0.44 |
| 130 | EC862624 | 7.51 | 1.71 | 4.38 | 3.63 | 0.94 | 3.63 | 0.03 | 4 | 0 | 0 | 12.33 | 17.67 | 30.00 | 0.70 |
| 131 | IC616213 | 40.24 | 5.65 | 6.57 | 15.19 | 0.70 | 14.51 | 0.06 | 24 | 22 | 2 | 33.65 | 66.35 | 100.00 | 0.51 |
| 132 | EC862607 | 3.92 | 1.91 | 1.74 | 2.83 | 0.89 | 2.15 | 0.02 | 12 | 6 | 0 | 8.34 | 11.67 | 20.00 | 0.71 |
| 133 | IC616117 | 7.56 | 2.16 | 3.44 | 3.15 | 0.79 | 3.03 | 0.02 | 8 | 0 | 0 | 4.22 | 5.78 | 10.00 | 0.73 |
| 134 | EC862635 | 15.52 | 3.49 | 4.45 | 13.17 | 0.87 | 13.17 | 0.08 | 22 | 24 | 0 | 8.22 | 11.78 | 20.00 | 0.70 |
| 135 | EC862601 | 7.01 | 2.35 | 2.98 | 3.52 | 1.10 | 3.52 | 0.03 | 4 | 0 | 0 | 36.45 | 73.55 | 110.00 | 0.50 |
| 136 | IC616142 | 13.16 | 3.11 | 3.77 | 4.88 | 0.89 | 4.21 | 0.03 | 2 | 0 | 0 | 4.34 | 7.67 | 12.00 | 0.57 |
| 137 | IC616118 | 60.07 | 8.32 | 6.63 | 34.23 | 0.67 | 33.56 | 0.12 | 39 | 34 | 5 | 8.34 | 21.67 | 30.00 | 0.38 |
| 138 | IC616187 | 33.85 | 4.57 | 7.31 | 17.69 | 0.56 | 17.57 | 0.04 | 27 | 15 | 2 | 8.33 | 19.67 | 28.00 | 0.42 |
| 139 | IC616163 | 9.00 | 2.29 | 3.94 | 3.97 | 0.83 | 3.97 | 0.02 | 4 | 0 | 0 | 9.33 | 20.67 | 30.00 | 0.45 |
| 140 | EC15024 | 9.50 | 1.97 | 4.83 | 5.42 | 0.63 | 5.42 | 0.02 | 2 | 0 | 0 | 8.67 | 11.33 | 20.00 | 0.76 |
| 141 | IC616232 | 15.19 | 3.62 | 3.71 | 5.09 | 0.75 | 4.41 | 0.02 | 7 | 1 | 0 | 5.33 | 7.67 | 13.00 | 0.70 |
| 142 | EC15184 | 7.26 | 2.29 | 3.18 | 2.86 | 0.77 | 2.86 | 0.01 | 2 | 0 | 0 | 4.55 | 5.45 | 10.00 | 0.84 |
| 143 | IC616188 | 12.19 | 2.67 | 4.57 | 4.65 | 1.26 | 4.65 | 0.06 | 4 | 0 | 0 | 48.66 | 81.34 | 130.00 | 0.60 |
| 144 | IC616277 | 36.09 | 6.16 | 5.30 | 16.79 | 0.80 | 16.12 | 0.08 | 21 | 11 | 0 | 8.22 | 19.78 | 28.00 | 0.42 |
| 145 | IC616241 | 19.29 | 4.19 | 4.09 | 7.38 | 0.95 | 6.71 | 0.05 | 10 | 6 | 1 | 8.55 | 14.45 | 23.00 | 0.59 |
| 146 | IC616205 | 11.24 | 2.79 | 3.94 | 3.26 | 0.82 | 3.14 | 0.02 | 6 | 0 | 0 | 9.88 | 13.12 | 23.00 | 0.75 |
| 147 | EC862596 | 11.48 | 2.48 | 4.64 | 4.50 | 0.72 | 4.50 | 0.02 | 2 | 0 | 0 | 8.99 | 11.01 | 20.00 | 0.82 |
| 148 | EC862655 | 13.31 | 3.49 | 3.81 | 3.69 | 1.22 | 3.69 | 0.04 | 2 | 0 | 0 | 36.89 | 73.11 | 110.00 | 0.50 |
| 149 | EC862599 | 27.69 | 4.25 | 5.99 | 14.87 | 0.81 | 14.20 | 0.08 | 17 | 30 | 0 | 14.66 | 25.34 | 40.00 | 0.58 |
| 150 | IC616255-a | 40.46 | 4.32 | 8.85 | 13.38 | 0.50 | 12.71 | 0.03 | 19 | 9 | 0 | 22.14 | 36.86 | 59.00 | 0.60 |
| 151 | IC616173 | 8.24 | 2.16 | 3.75 | 3.41 | 1.01 | 3.29 | 0.03 | 4 | 0 | 0 | 38.45 | 60.29 | 98.74 | 0.64 |
| 152 | EC862676 | 4.49 | 1.33 | 3.37 | 3.06 | 0.83 | 3.06 | 0.02 | 6 | 2 | 0 | 4.10 | 5.90 | 10.00 | 0.69 |
| 153 | EC862614 | 6.00 | 1.52 | 3.94 | 2.92 | 1.16 | 2.92 | 0.03 | 2 | 0 | 0 | 49.55 | 64.70 | 114.25 | 0.77 |
| 154 | EC862644 | 5.93 | 1.65 | 3.33 | 3.38 | 1.24 | 2.70 | 0.04 | 6 | 0 | 0 | 39.77 | 89.23 | 129.00 | 0.45 |
| 155 | IC616201 | 26.06 | 4.06 | 6.41 | 8.58 | 0.85 | 8.58 | 0.05 | 11 | 3 | 0 | 15.33 | 30.67 | 46.00 | 0.50 |
| 156 | IC616168 | 17.47 | 3.62 | 4.83 | 6.84 | 0.98 | 6.84 | 0.05 | 18 | 4 | 0 | 8.20 | 14.80 | 23.00 | 0.55 |
| 157 | EC862660 | 43.13 | 4.51 | 9.04 | 19.67 | 0.58 | 19.00 | 0.05 | 33 | 13 | 1 | 5.66 | 14.34 | 20.00 | 0.40 |
| 158 | IC616257 | 25.92 | 4.32 | 5.49 | 5.01 | 0.83 | 4.34 | 0.03 | 20 | 0 | 0 | 46.34 | 103.67 | 150.00 | 0.45 |
| 159 | IC616260 | 53.35 | 5.65 | 9.34 | 20.29 | 0.59 | 20.17 | 0.06 | 27 | 13 | 0 | 8.67 | 11.34 | 20.00 | 0.76 |
| 160 | IC616269 | 20.09 | 3.37 | 5.97 | 6.05 | 0.89 | 6.05 | 0.04 | 12 | 0 | 0 | 18.22 | 61.78 | 80.00 | 0.29 |
| 161 | EC862643 | 19.92 | 4.83 | 4.13 | 4.68 | 1.05 | 4.68 | 0.04 | 6 | 0 | 0 | 29.88 | 84.12 | 114.00 | 0.36 |
| 162 | IC616124 | 19.99 | 3.87 | 4.66 | 3.91 | 0.85 | 3.24 | 0.02 | 6 | 2 | 0 | 7.55 | 18.45 | 26.00 | 0.41 |
| 163 | IC61617 | 32.04 | 3.87 | 7.77 | 16.97 | 0.67 | 16.30 | 0.06 | 30 | 18 | 2 | 14.34 | 25.67 | 40.00 | 0.56 |
| 164 | IC616141 | 34.32 | 4.45 | 7.63 | 11.57 | 0.62 | 11.45 | 0.04 | 23 | 15 | 0 | 15.34 | 29.66 | 45.00 | 0.52 |
| 165 | IC616178 | 12.73 | 2.60 | 4.89 | 5.07 | 1.02 | 5.07 | 0.04 | 2 | 0 | 0 | 9.22 | 16.78 | 26.00 | 0.55 |
| 166 | IC616219 | 6.86 | 2.92 | 2.35 | 2.15 | 1.11 | 2.15 | 0.02 | 6 | 0 | 0 | 37.55 | 81.45 | 119.00 | 0.46 |
| 167 | EC862613 | 12.39 | 2.54 | 4.47 | 9.25 | 0.83 | 8.58 | 0.05 | 16 | 10 | 0 | 9.22 | 20.78 | 30.00 | 0.44 |
| 168 | IC616251 | 27.36 | 3.68 | 7.43 | 15.87 | 0.74 | 15.87 | 0.07 | 27 | 35 | 0 | 8.77 | 21.23 | 30.00 | 0.41 |
| 169 | EC862664 | 31.75 | 3.94 | 8.06 | 13.29 | 0.85 | 13.29 | 0.08 | 34 | 18 | 1 | 20.11 | 29.89 | 50.00 | 0.67 |
| 170 | IC616147 | 5.58 | 1.97 | 2.50 | 1.80 | 1.14 | 1.13 | 0.02 | 5 | 1 | 0 | 21.30 | 58.70 | 80.00 | 0.36 |
| 171 | IC616144 | 9.53 | 2.92 | 2.82 | 4.66 | 0.87 | 3.98 | 0.03 | 6 | 0 | 0 | 24.22 | 35.78 | 60.00 | 0.68 |
| 172 | EC15181 | 11.86 | 3.43 | 3.37 | 4.08 | 0.79 | 3.96 | 0.02 | 2 | 0 | 0 | 26.55 | 43.45 | 70.00 | 0.61 |
| 173 | EC15158 | 7.39 | 2.48 | 2.98 | 2.81 | 1.34 | 2.81 | 0.04 | 2 | 0 | 0 | 36.89 | 60.99 | 97.88 | 0.60 |
| 174 | EC15026 | 14.88 | 2.86 | 5.21 | 10.63 | 0.81 | 10.63 | 0.06 | 20 | 19 | 0 | 9.55 | 20.45 | 30.00 | 0.47 |
| 175 | EC862668 | 22.41 | 3.37 | 6.18 | 11.39 | 0.74 | 10.72 | 0.05 | 25 | 7 | 0 | 25.33 | 54.67 | 80.00 | 0.46 |
| 176 | IC616155 | 33.20 | 4.45 | 6.95 | 12.16 | 0.58 | 11.49 | 0.03 | 24 | 14 | 0 | 8.66 | 16.34 | 25.00 | 0.53 |
| 177 | EC862687 | 8.22 | 2.48 | 3.25 | 2.87 | 1.33 | 2.75 | 0.04 | 2 | 0 | 0 | 38.99 | 85.01 | 124.00 | 0.46 |
| 178 | IC616262 | 41.92 | 4.00 | 10.48 | 14.05 | 0.51 | 14.05 | 0.03 | 15 | 11 | 0 | 16.32 | 33.68 | 50.00 | 0.48 |
| 179 | EC862652 | 20.18 | 3.49 | 5.78 | 6.75 | 0.98 | 6.75 | 0.05 | 15 | 1 | 0 | 9.22 | 18.78 | 28.00 | 0.49 |
| 180 | IC616183 | 16.18 | 3.49 | 4.15 | 6.51 | 0.65 | 5.84 | 0.02 | 11 | 5 | 0 | 8.22 | 17.78 | 26.00 | 0.46 |
| 181 | IC616207 | 9.25 | 2.35 | 3.94 | 3.33 | 1.05 | 3.33 | 0.03 | 8 | 0 | 0 | 54.22 | 80.78 | 135.00 | 0.67 |
| 182 | EC862661 | 7.85 | 2.10 | 3.75 | 2.70 | 1.41 | 2.70 | 0.04 | 5 | 1 | 0 | 38.77 | 85.23 | 124.00 | 0.45 |
| 183 | IC616112 | 5.54 | 2.79 | 1.55 | 3.07 | 0.97 | 2.40 | 0.02 | 4 | 0 | 0 | 4.50 | 7.50 | 12.00 | 0.60 |
| 184 | EC862622 | 3.46 | 2.41 | 1.04 | 2.55 | 1.06 | 1.88 | 0.02 | 2 | 0 | 0 | 3.22 | 6.78 | 10.00 | 0.48 |
| 185 | IC616153 | 11.20 | 2.29 | 4.83 | 4.74 | 0.81 | 4.62 | 0.02 | 8 | 0 | 0 | 9.22 | 26.78 | 36.00 | 0.34 |
| 186 | IC616115 | 55.75 | 5.83 | 9.56 | 17.86 | 0.39 | 17.86 | 0.02 | 12 | 23 | 4 | 25.11 | 39.89 | 65.00 | 0.63 |
| 187 | IC616209 | 36.03 | 3.92 | 9.18 | 23.92 | 0.47 | 23.92 | 0.04 | 21 | 25 | 4 | 16.33 | 25.67 | 42.00 | 0.64 |
| 188 | EC862690 | 56.63 | 5.41 | 9.92 | 15.64 | 0.46 | 14.97 | 0.03 | 8 | 14 | 2 | 26.55 | 61.45 | 88.00 | 0.43 |
| 189 | IC616211 | 46.05 | 5.98 | 7.14 | 32.37 | 0.55 | 31.69 | 0.08 | 16 | 51 | 2 | 24.30 | 41.70 | 66.00 | 0.58 |
| 190 | IC616131 | 29.61 | 2.93 | 10.02 | 21.40 | 0.47 | 21.28 | 0.04 | 8 | 16 | 6 | 8.66 | 23.34 | 32.00 | 0.37 |
| 191 | IC616229 | 78.01 | 7.39 | 10.55 | 12.86 | 0.50 | 12.86 | 0.03 | 16 | 10 | 0 | 36.55 | 53.45 | 90.00 | 0.68 |
| 192 | IC616223 | 19.28 | 3.05 | 6.32 | 25.99 | 0.59 | 25.99 | 0.07 | 31 | 39 | 3 | 12.20 | 17.80 | 30.00 | 0.69 |
| 193 | IC616114 | 58.40 | 5.99 | 9.18 | 6.74 | 0.48 | 6.07 | 0.01 | 2 | 0 | 0 | 34.50 | 55.50 | 90.00 | 0.62 |
| 194 | IC616127 | 12.03 | 4.01 | 3.00 | 11.78 | 0.51 | 11.78 | 0.02 | 8 | 2 | 0 | 15.66 | 44.34 | 60.00 | 0.35 |
| 195 | IC616185 | 35.43 | 4.47 | 7.92 | 6.02 | 0.65 | 6.02 | 0.02 | 10 | 4 | 0 | 16.54 | 23.46 | 40.00 | 0.71 |
| 196 | IC616268 | 75.81 | 7.42 | 9.64 | 18.25 | 0.56 | 17.58 | 0.04 | 15 | 25 | 2 | 14.87 | 35.13 | 50.00 | 0.42 |
| 197 | EC862646 | 36.57 | 4.17 | 8.27 | 18.75 | 0.45 | 18.08 | 0.03 | 11 | 17 | 1 | 12.55 | 17.45 | 30.00 | 0.72 |
| 198 | IC616152 | 16.93 | 2.84 | 5.88 | 9.33 | 0.47 | 9.21 | 0.02 | 5 | 3 | 0 | 9.22 | 15.78 | 25.00 | 0.58 |
| 199 | EC862585 | 34.00 | 4.32 | 7.87 | 14.80 | 0.42 | 14.80 | 0.02 | 14 | 22 | 4 | 9.66 | 26.34 | 36.00 | 0.37 |
| 200 | IC616271 | 58.53 | 5.49 | 10.67 | 17.19 | 0.45 | 17.19 | 0.03 | 12 | 18 | 1 | 26.55 | 43.45 | 70.00 | 0.61 |
| 201 | EC862594 | 133.38 | 8.74 | 14.67 | 15.41 | 0.63 | 14.73 | 0.05 | 8 | 24 | 0 | 68.22 | 96.78 | 165.00 | 0.70 |
| 202 | IC616247 | 95.39 | 7.16 | 12.74 | 56.89 | 0.53 | 56.22 | 0.13 | 46 | 62 | 5 | 56.22 | 79.78 | 136.00 | 0.70 |
| 203 | IC616099 | 53.45 | 5.64 | 9.38 | 43.94 | 0.54 | 43.82 | 0.10 | 32 | 42 | 2 | 9.56 | 35.44 | 45.00 | 0.27 |
| 204 | IC616245 | 69.99 | 5.74 | 12.19 | 26.08 | 0.58 | 26.08 | 0.07 | 28 | 28 | 0 | 18.22 | 35.78 | 54.00 | 0.51 |
| 205 | EC15137 | 79.19 | 5.69 | 13.92 | 22.62 | 0.62 | 22.62 | 0.07 | 27 | 25 | 0 | 24.00 | 46.00 | 70.00 | 0.52 |
| 206 | IC616100 | 98.86 | 7.37 | 12.84 | 38.10 | 0.54 | 37.42 | 0.09 | 29 | 41 | 1 | 56.33 | 99.67 | 156.00 | 0.57 |
| 207 | IC616237 | 91.28 | 6.65 | 13.72 | 36.77 | 0.49 | 36.77 | 0.07 | 33 | 59 | 6 | 42.22 | 85.78 | 128.00 | 0.49 |
| 208 | EC15040 | 87.10 | 7.62 | 11.43 | 33.01 | 0.52 | 33.01 | 0.07 | 27 | 44 | 4 | 17.66 | 22.34 | 40.00 | 0.79 |
| 209 | IC616259 | 87.77 | 7.77 | 10.71 | 28.25 | 0.55 | 27.57 | 0.07 | 31 | 21 | 0 | 23.11 | 56.89 | 80.00 | 0.41 |
| 210 | IC616109 | 116.17 | 7.82 | 14.26 | 40.19 | 0.45 | 39.52 | 0.06 | 34 | 46 | 8 | 45.34 | 104.66 | 150.00 | 0.43 |
| 211 | EC862670 | 83.28 | 5.74 | 14.41 | 60.35 | 0.47 | 60.23 | 0.10 | 58 | 70 | 12 | 24.00 | 41.00 | 65.00 | 0.59 |
| 212 | EC862603 | 96.74 | 7.47 | 12.95 | 32.16 | 0.52 | 32.16 | 0.07 | 40 | 30 | 2 | 51.22 | 84.78 | 136.00 | 0.60 |
| 213 | EC862685 | 42.39 | 3.81 | 11.13 | 34.83 | 0.51 | 34.83 | 0.07 | 48 | 26 | 2 | 6.22 | 13.78 | 20.00 | 0.45 |
| 214 | IC616239 | 38.27 | 4.01 | 9.03 | 36.61 | 0.45 | 35.93 | 0.06 | 68 | 42 | 5 | 3.11 | 6.89 | 10.00 | 0.45 |
| 215 | EC15179 | 72.86 | 7.06 | 9.74 | 17.94 | 0.59 | 17.27 | 0.05 | 22 | 22 | 0 | 14.33 | 35.67 | 50.00 | 0.40 |
| 216 | IC616265 | 55.43 | 5.33 | 10.29 | 29.52 | 0.57 | 29.40 | 0.07 | 20 | 43 | 1 | 9.55 | 20.45 | 30.00 | 0.47 |
| 217 | EC862663 | 78.66 | 6.45 | 12.19 | 20.72 | 0.48 | 20.72 | 0.04 | 25 | 17 | 0 | 9.77 | 20.23 | 30.00 | 0.48 |
| 218 | IC616189 | 66.99 | 5.59 | 11.99 | 40.40 | 0.45 | 40.40 | 0.06 | 46 | 32 | 1 | 23.11 | 56.89 | 80.00 | 0.41 |
| 219 | IC616111 | 58.37 | 5.59 | 9.89 | 20.35 | 0.56 | 19.67 | 0.05 | 50 | 20 | 1 | 21.33 | 30.67 | 52.00 | 0.70 |
| 220 | IC616107 | 95.54 | 6.25 | 15.29 | 16.76 | 0.52 | 16.76 | 0.04 | 39 | 20 | 1 | 43.20 | 68.80 | 112.00 | 0.63 |
| 221 | IC616223 | 88.44 | 7.32 | 12.09 | 39.58 | 0.51 | 39.58 | 0.08 | 61 | 35 | 6 | 35.44 | 63.56 | 99.00 | 0.56 |
| 222 | IC616132 | 81.89 | 6.55 | 11.92 | 22.51 | 0.62 | 21.84 | 0.07 | 33 | 17 | 1 | 46.22 | 63.78 | 110.00 | 0.72 |
| 223 | EC15168 | 70.23 | 5.79 | 11.57 | 25.30 | 0.46 | 24.63 | 0.04 | 32 | 18 | 4 | 26.22 | 33.78 | 60.00 | 0.78 |
| 224 | IC616121 | 87.70 | 8.43 | 10.29 | 27.88 | 0.51 | 27.77 | 0.06 | 26 | 29 | 6 | 21.33 | 58.67 | 80.00 | 0.36 |
| 225 | EC15020 | 82.22 | 6.86 | 11.99 | 40.61 | 0.52 | 40.61 | 0.09 | 52 | 40 | 2 | 22.67 | 54.34 | 77.00 | 0.42 |
| 226 | IC616193 | 72.46 | 6.10 | 11.89 | 36.85 | 0.49 | 36.85 | 0.07 | 63 | 41 | 2 | 22.10 | 43.90 | 66.00 | 0.50 |
| 227 | EC862620 | 91.70 | 7.98 | 10.91 | 30.80 | 0.48 | 30.13 | 0.06 | 27 | 25 | 3 | 35.66 | 84.34 | 120.00 | 0.42 |
| 228 | EC862616 | 103.07 | 7.62 | 12.94 | 20.91 | 0.63 | 20.24 | 0.06 | 38 | 20 | 0 | 54.22 | 114.78 | 169.00 | 0.47 |
| 229 | EC862627 | 92.44 | 6.20 | 14.82 | 34.35 | 0.61 | 34.23 | 0.10 | 50 | 40 | 2 | 14.00 | 26.00 | 40.00 | 0.54 |
| 230 | IC616264 | 65.74 | 4.78 | 13.77 | 33.48 | 0.63 | 33.48 | 0.10 | 52 | 67 | 0 | 32.01 | 54.99 | 87.00 | 0.58 |
| 231 | IC616226 | 96.39 | 7.62 | 12.65 | 24.26 | 0.59 | 24.26 | 0.07 | 52 | 30 | 1 | 26.55 | 83.45 | 110.00 | 0.32 |
| 232 | EC862677 | 80.51 | 7.26 | 10.50 | 29.48 | 0.55 | 28.81 | 0.07 | 35 | 27 | 0 | 26.33 | 41.67 | 68.00 | 0.63 |
| 233 | EC862632 | 111.51 | 7.57 | 14.73 | 28.63 | 0.59 | 28.63 | 0.08 | 30 | 30 | 3 | 59.66 | 124.34 | 184.00 | 0.48 |
| 234 | IC61625 | 109.05 | 7.06 | 15.44 | 47.80 | 0.61 | 47.80 | 0.14 | 60 | 79 | 7 | 68.22 | 95.78 | 164.00 | 0.71 |
| 235 | IC616192 | 39.98 | 4.52 | 8.32 | 37.07 | 0.56 | 36.40 | 0.09 | 40 | 63 | 6 | 15.66 | 24.34 | 40.00 | 0.64 |
| 236 | IC616149 | 78.02 | 6.15 | 12.13 | 27.85 | 0.50 | 27.18 | 0.05 | 52 | 31 | 2 | 21.03 | 58.97 | 80.00 | 0.36 |
| 237 | IC616175 | 87.25 | 5.64 | 15.37 | 34.70 | 0.47 | 34.58 | 0.06 | 75 | 29 | 2 | 21.30 | 28.70 | 50.00 | 0.74 |
| 238 | IC862636 | 103.67 | 7.06 | 14.68 | 34.93 | 0.49 | 34.93 | 0.07 | 39 | 40 | 7 | 2.60 | 7.40 | 10.00 | 0.35 |
| 239 | IC616221 | 82.80 | 6.20 | 13.36 | 27.20 | 0.51 | 27.20 | 0.06 | 39 | 29 | 4 | 21.30 | 28.70 | 50.00 | 0.74 |
| 240 | IC616164 | 107.76 | 7.57 | 13.65 | 35.88 | 0.51 | 35.21 | 0.07 | 42 | 32 | 2 | 21.00 | 39.00 | 60.00 | 0.54 |
| 241 | IC616166 | 67.28 | 5.89 | 10.86 | 45.19 | 0.55 | 44.52 | 0.11 | 49 | 57 | 4 | 19.55 | 50.45 | 70.00 | 0.39 |
| 242 | IC616244 | 60.09 | 5.13 | 11.62 | 26.95 | 0.54 | 26.83 | 0.06 | 37 | 17 | 1 | 8.99 | 25.01 | 34.00 | 0.36 |
| 243 | IC616130 | 56.76 | 5.94 | 9.55 | 18.69 | 0.53 | 18.69 | 0.04 | 50 | 28 | 0 | 26.55 | 53.45 | 80.00 | 0.50 |
| 244 | EC862648 | 23.66 | 2.44 | 9.70 | 27.63 | 0.54 | 27.63 | 0.06 | 22 | 16 | 1 | 8.77 | 11.23 | 20.00 | 0.78 |
| 245 | IC616120 | 61.45 | 5.99 | 9.69 | 19.17 | 0.45 | 18.50 | 0.03 | 31 | 19 | 4 | 24.67 | 63.34 | 88.00 | 0.39 |
| 246 | EC862604 | 61.63 | 8.48 | 7.26 | 19.53 | 0.52 | 19.53 | 0.04 | 31 | 24 | 3 | 36.55 | 50.45 | 87.00 | 0.72 |
| 247 | EC862656 | 67.40 | 4.62 | 14.58 | 21.33 | 0.58 | 21.33 | 0.06 | 27 | 50 | 4 | 13.20 | 16.80 | 30.00 | 0.79 |
| 248 | IC616126 | 63.94 | 5.44 | 11.21 | 23.90 | 0.51 | 23.23 | 0.05 | 36 | 27 | 1 | 11.20 | 18.80 | 30.00 | 0.60 |
| 249 | IC616254 | 52.81 | 4.37 | 11.57 | 14.80 | 0.58 | 14.13 | 0.04 | 15 | 9 | 0 | 10.25 | 19.75 | 30.00 | 0.52 |
| 250 | EC862618 | 40.76 | 4.06 | 9.94 | 28.63 | 0.54 | 28.51 | 0.07 | 64 | 32 | 1 | 14.67 | 25.34 | 40.00 | 0.58 |
| 251 | EC15171 | 52.68 | 5.49 | 9.60 | 16.22 | 0.56 | 16.22 | 0.04 | 34 | 12 | 0 | 24.55 | 43.45 | 68.00 | 0.57 |
| 252 | IC616106 | 57.97 | 4.88 | 11.89 | 22.81 | 0.53 | 22.81 | 0.05 | 59 | 32 | 1 | 15.66 | 14.34 | 30.00 | 1.09 |
| 253 | IC616255-B | 82.94 | 6.86 | 11.52 | 21.59 | 0.51 | 20.91 | 0.04 | 45 | 24 | 0 | 24.66 | 43.34 | 68.00 | 0.57 |
| 254 | IC616203 | 125.33 | 7.98 | 15.13 | 27.31 | 0.55 | 26.63 | 0.06 | 43 | 23 | 2 | 65.45 | 94.55 | 160.00 | 0.69 |
| 255 | IC616246 | 83.24 | 5.64 | 14.66 | 42.70 | 0.50 | 42.58 | 0.08 | 41 | 29 | 3 | 21.66 | 34.34 | 56.00 | 0.63 |
| 256 | IC616276 | 109.83 | 6.76 | 16.26 | 31.16 | 0.57 | 31.16 | 0.08 | 46 | 24 | 2 | 67.66 | 102.34 | 170.00 | 0.66 |
| 257 | IC616253 | 98.26 | 8.48 | 11.58 | 40.14 | 0.49 | 40.14 | 0.08 | 136 | 44 | 5 | 55.40 | 74.60 | 130.00 | 0.74 |
| 258 | IC616266 | 66.69 | 5.84 | 10.86 | 28.82 | 0.58 | 28.15 | 0.08 | 48 | 55 | 1 | 24.66 | 41.34 | 66.00 | 0.60 |
| 259 | EC862589 | 112.32 | 7.92 | 14.17 | 36.40 | 0.49 | 36.40 | 0.07 | 53 | 43 | 8 | 40.67 | 89.34 | 130.00 | 0.46 |
| 260 | IC616234 | 58.54 | 5.38 | 10.87 | 21.11 | 0.55 | 21.11 | 0.05 | 52 | 14 | 1 | 20.55 | 59.45 | 80.00 | 0.35 |
| 261 | EC862675 | 54.35 | 5.23 | 9.84 | 15.70 | 0.68 | 15.03 | 0.06 | 36 | 10 | 2 | 15.66 | 27.34 | 43.00 | 0.57 |
| 262 | IC616103 | 59.90 | 4.93 | 11.62 | 19.32 | 0.62 | 18.65 | 0.06 | 29 | 23 | 1 | 17.88 | 33.12 | 51.00 | 0.54 |
| 263 | IC616225 | 64.96 | 6.60 | 9.74 | 16.80 | 0.59 | 16.68 | 0.05 | 82 | 12 | 0 | 19.66 | 35.34 | 55.00 | 0.56 |
| 264 | IC616270 | 47.34 | 4.22 | 11.23 | 19.83 | 0.58 | 19.83 | 0.05 | 30 | 12 | 1 | 9.89 | 20.11 | 30.00 | 0.49 |
| 265 | IC616102 | 66.19 | 5.23 | 12.65 | 18.25 | 0.53 | 18.25 | 0.04 | 34 | 27 | 1 | 15.66 | 34.34 | 50.00 | 0.46 |
| 266 | EC862598 | 13.84 | 3.86 | 3.09 | 25.24 | 0.59 | 24.56 | 0.07 | 72 | 26 | 0 | 76.55 | 133.45 | 210.00 | 0.57 |
| 267 | IC616243 | 67.40 | 6.15 | 10.40 | 3.96 | 0.95 | 3.29 | 0.03 | 9 | 1 | 0 | 48.66 | 71.34 | 120.00 | 0.68 |
| 268 | IC616133 | 40.53 | 4.47 | 8.97 | 18.94 | 0.56 | 18.82 | 0.05 | 26 | 19 | 0 | 24.31 | 45.69 | 70.00 | 0.53 |
| 269 | IC616104 | 38.13 | 3.81 | 10.01 | 21.43 | 0.52 | 21.43 | 0.05 | 50 | 32 | 1 | 19.22 | 30.78 | 50.00 | 0.62 |
| 270 | IC616236 | 72.95 | 5.59 | 13.06 | 15.75 | 0.53 | 15.75 | 0.04 | 78 | 16 | 0 | 16.55 | 33.45 | 50.00 | 0.49 |
| 271 | IC616217 | 54.71 | 4.98 | 10.45 | 28.03 | 0.57 | 27.35 | 0.07 | 101 | 43 | 2 | 21.22 | 38.78 | 60.00 | 0.55 |
| 272 | EC862674 | 64.10 | 6.10 | 10.52 | 19.24 | 0.56 | 19.24 | 0.05 | 34 | 16 | 0 | 46.22 | 63.78 | 110.00 | 0.72 |
| 273 | IC616180 | 57.29 | 6.10 | 9.40 | 39.14 | 0.53 | 39.14 | 0.09 | 39 | 48 | 5 | 14.33 | 18.67 | 33.00 | 0.77 |
| 274 | EC862606 | 63.84 | 6.35 | 9.49 | 13.79 | 0.53 | 13.12 | 0.03 | 28 | 12 | 1 | 14.67 | 29.34 | 44.00 | 0.50 |
| 275 | IC616206 | 71.79 | 5.94 | 11.52 | 13.28 | 0.51 | 12.61 | 0.03 | 17 | 9 | 0 | 34.89 | 55.11 | 90.00 | 0.63 |
| 276 | EC862666 | 102.50 | 8.38 | 12.12 | 29.80 | 0.48 | 29.68 | 0.05 | 67 | 31 | 3 | 46.89 | 65.11 | 112.00 | 0.72 |
| 277 | IC616184 | 113.09 | 8.43 | 13.41 | 36.62 | 0.50 | 36.62 | 0.07 | 181 | 36 | 1 | 58.66 | 85.34 | 144.00 | 0.69 |
| 278 | EC15144 | 49.98 | 5.44 | 9.19 | 21.89 | 0.54 | 21.89 | 0.05 | 42 | 27 | 1 | 15.66 | 24.34 | 40.00 | 0.64 |
| 279 | IC616210 | 82.59 | 6.86 | 11.47 | 13.79 | 0.51 | 13.11 | 0.03 | 56 | 22 | 0 | 14.56 | 35.44 | 50.00 | 0.41 |
| 280 | IC616249 | 83.36 | 6.81 | 11.67 | 37.23 | 0.46 | 36.56 | 0.06 | 73 | 37 | 9 | 16.55 | 32.45 | 49.00 | 0.51 |
| 281 | IC616108 | 92.14 | 6.60 | 13.85 | 33.32 | 0.51 | 33.20 | 0.07 | 46 | 30 | 4 | 45.89 | 104.11 | 150.00 | 0.44 |
| 282 | IC616101 | 69.58 | 6.55 | 10.62 | 45.18 | 0.52 | 45.18 | 0.10 | 161 | 36 | 8 | 23.11 | 66.89 | 90.00 | 0.35 |
| 283 | EC862693 | 64.41 | 5.28 | 12.19 | 23.79 | 0.55 | 23.79 | 0.06 | 128 | 32 | 1 | 24.11 | 35.89 | 60.00 | 0.67 |
| 284 | EC862605 | 33.61 | 4.11 | 7.66 | 35.84 | 0.53 | 35.17 | 0.08 | 59 | 38 | 4 | 3.22 | 6.78 | 10.00 | 0.47 |
| 285 | IC15252 | 89.29 | 8.79 | 10.16 | 17.13 | 0.52 | 17.13 | 0.04 | 18 | 20 | 0 | 36.55 | 53.45 | 90.00 | 0.68 |
| 286 | EC15046 | 87.48 | 5.74 | 15.24 | 25.13 | 0.50 | 25.13 | 0.05 | 64 | 30 | 0 | 24.66 | 45.34 | 70.00 | 0.54 |
| 287 | EC862608 | 84.17 | 5.94 | 13.60 | 22.80 | 0.49 | 22.12 | 0.04 | 32 | 14 | 0 | 14.22 | 25.78 | 40.00 | 0.55 |
| 288 | EC15229 | 79.60 | 7.82 | 9.59 | 30.10 | 0.58 | 29.42 | 0.08 | 62 | 30 | 1 | 15.87 | 34.13 | 50.00 | 0.46 |
| 289 | EC15006 | 86.28 | 6.30 | 13.60 | 25.33 | 0.45 | 25.21 | 0.04 | 139 | 35 | 2 | 17.88 | 42.12 | 60.00 | 0.42 |
| 290 | EC862587 | 54.25 | 4.62 | 11.73 | 22.76 | 0.47 | 22.76 | 0.04 | 39 | 19 | 0 | 19.44 | 30.56 | 50.00 | 0.64 |
| 291 | IC616137 | 45.65 | 4.47 | 10.21 | 21.89 | 0.50 | 21.89 | 0.04 | 42 | 18 | 0 | 17.88 | 52.12 | 70.00 | 0.34 |
| 292 | EC862639 | 49.73 | 5.33 | 8.78 | 15.41 | 0.59 | 14.74 | 0.04 | 27 | 15 | 0 | 16.55 | 28.45 | 45.00 | 0.58 |
| 293 | IC616272 | 43.64 | 5.38 | 7.56 | 14.91 | 0.71 | 14.24 | 0.06 | 39 | 11 | 0 | 19.99 | 26.01 | 46.00 | 0.77 |
| 294 | IC616165 | 37.63 | 4.67 | 7.96 | 14.00 | 0.66 | 13.88 | 0.05 | 20 | 10 | 0 | 9.88 | 25.12 | 35.00 | 0.39 |
| 295 | EC862626 | 50.14 | 6.45 | 7.77 | 20.29 | 0.55 | 20.29 | 0.05 | 17 | 39 | 2 | 19.89 | 50.11 | 70.00 | 0.40 |
| 296 | IC616220 | 49.84 | 4.42 | 11.28 | 28.57 | 0.54 | 28.57 | 0.07 | 76 | 37 | 2 | 3.56 | 6.44 | 10.00 | 0.55 |

(PRL-PrimaryRoot length, TPA: Total project area ,TSA: Total surface area ,TRL: Total root length , ARD : Average Root Diameter, LPV: Length per volume , TRV: Total Root Volume , TRT : Total Root Tips, TRF: Total Root Forks, TRC : Total root crossings, RDW: Root dry weight, SDW: Seed Dry weight , TDW: Total Dry weight.RSR: Root to shoot ratio.)
