# Supplementary material for: Assessment of root phenotypes in mungbean mini-core collection (MMC) from the World Vegetable Center (AVRDC) Taiwan
Source: PLoS One. 2021 Mar 4;16(3):e0247810. doi: 10.1371/journal.pone.0247810 (PMC7932546; doi:10.1371/journal.pone.0247810)
Supplement: Attachment — Submitted filename: Response to reviwer .docx [file pone.0247810.s003.docx]

**Response to Reviewer 1:**

|  | **Concerns** | **Reply** |
| --- | --- | --- |
| 1 | Line 19 – root key root traits change to key root traits | **Changed to key root traits** |
| 2 | Line 56 – Modify the sentence | **As per suggestions sentences modified and rewritten to convey the details of phenotyping/screening and imaging techniques.**  In order to examine root features, several phenotyping techniques were documented, particularly hydroponic systems utilizing growth bags (or germination sheet) [52-54], agar-plate and aeroponic systems [55], soil rhizotrons [56-58], deep column methods [59], transparent containers [60], PVC pipes (columns) and glass-walled rhizoboxes filled with soil, but these methods are costly, require considerable labour and large area for phenotyping larger genotype sets [61-63]. The unique semi-hydroponic phenotyping system[64] was established to assess the heterogeneity of the root trait in the narrow-leaf lupin (*Lupinus angustifolius* L.) core set[65-66].The same technique was used for root phenotyping in chickpea (*Cicer arietinum* L.)[67], maize (*Zea mays* L.) and barley (*Hordeum vulgare* L.)[68]. This unique semi-hydroponic technique was modified to suit the purpose to screen a mungbean mini-core collection for root traits.  The use of digital imaging and software tools for root image analysis is an innovative and efficient way to accurately assess root traits [69-71]. Several software programmes are available to extract two-dimensional root morphology traits. This varies from the manual root label DART [70], commercially available software and semi-automated root analysis tool WinRhizo^TM^ (Regent Instruments, Québec, Canada) (Pro, 2004) and EzRhizo[72], freely usable, fully integrated and automated SmartRoot [73] software for small root systems. |
| 3 | **Introduction:** Add some details showing the updates/stubs of root system architecture analysis and application of root traits in legume improvement (there are several studies in soybean, chickpea and other legumes). | **As suggested, specifics are added to the root system architecture research and its application to improve legumes (soybean, chickpea and other legumes).**  Root system architecture is seen as the main factor for efficient water absorption and therefore for maintaining productivity in conditions of drought [23]. Root features are commonly known as the root system architecture (RSA), which refers to the form of the roots and their physical space. With its ability to obtain more water from the soil, a deeper and more proliferative RSA is also capable of avoiding water deficit conditions.  Roots begin to spread into deeper soil layers as plants encounter water deficit stress [24-25].The root diameter and distribution of root conductivity-regulating metaxyl vessels are also documented to provide drought tolerance in food grain legumes [26]. Thicker roots prefer to penetrate deeper into soil layers [27].  The key determinants of proliferative rooting are the initiation and elongation of the lateral root, which usually refers to the sum of the lateral root, root volume, root surface and root length density. Proliferative and deeper roots have increased capacity for water absorption in water-deficient soils [28-29]. The ideal root phenotypes under water deficit situations, in food grain legumes include root surface area [30], root length [31], deeper and proliferative roots [32-33] in soybean (*Glycine max* L.), root diameter in cowpea (*Vigna unguiculata* L.) [34], root length in pea (*Pisum sativum* L.) [35], basal root angle in common bean (*Phaseolus vulgaris* L.) [36], and rooting depth, root surface area, root length density [37] and proliferative & deeper roots [38-39] in chickpea (*Cicer arietinum* L.). Other crops which have benefited from ideal root phenotypes like proliferative and deep root traits under drought tolerance include rice (*Oryza sativa*), [40-41] , maize (*Zea mays*) [42-43] , barley (*Hordeum vulgare*) [44] , wheat (*Tritcum aestivum)* [45-46] |
| 3 | **Discussion:** This section need to be improved. At present it is descriptive with repeating contents from the results section. Need more supporting evidences and take home messages based on the findings. | **As per the comments, this segment has been revised and supporting evidence has been added to make it meaning full with take home messages.**  Across both breeding programmes and scientific research, the quest for root characteristics has intensified, offering improvement in the acquisition of resources and tolerance to abiotic stress including heat and drought stress, especially in resource-poor environments. Advancement was delayed due to problems in effective and accurate root trait phenotyping in large germplasm panels [49-50, 87]. In a mini-core set of 296 mung bean genotypes, the use of a modified semi-hydroponic phenotyping system saved time and space for phenotyping and provided access to substantial variation of root traits.  The novel method of semi-hydroponic phenotyping [64] has been modified to screen and explore the genetic variation of different root characters at the early vegetative stage of a mungbean mini-core set (Table 2, Supplementary Table 1).Previously, the semi-hydroponic phenotyping platform provided quality data for the wild narrow leaf lupin[65], core collections of lupin[66], and chickpea [67] to assess the genetic basis of variation in root characteristics.  The mungbean root system is similar those of other dicotyledonous species including Arabidopsis, Medicago, and other legumes like chickpea and develops through consecutive branch/lateral root orders from a primary root that emerges from the embryo[88]. The volume and size of lateral branches/roots is an important contributor for the growth and development of food grain legumes [89].  Depth of rooting and density of root branches are essential architectural features that directly affect water and nutrients acquisition in the soil strata[90]. The PRL of the deep-rooting genotype EC862594 was more than twice as high than the average value of the whole germplasm set and genotype EC 862670 had a more than 3-fold TRL than the average, corroborating the trait diversity present in the mini-core collection (Supplementary Table 2). Another aspect that affects deep rooting is root penetrability and root thickness or root diameter [91]. The thicker roots prefer to go deeper into the soil in order to obtain water from deeper soil layers [27].Research shows that in chickpea there is a strong association between prolific root system and the development of grain in terminal drought situations [38].In the case of total surface area, the best genotype IC 616276 only had about 20% more TSA than the average. This suggests that the TSA exhibited lower variations in the Mungbean mini-core collection. (Table 5 and Supplementary Table 2).  Proliferative rooting is primarily characterised by the initiation and elongation of lateral roots, which usually refers to the number of the lateral root, the root length density (RLD), and the root volume and the root surface area. Proliferative roots have a considerably large water absorption potential in water deficit soils. In environments with water scarcity, lines with higher RLD showed clearly improved yield and drought-tolerance-related performance [92].  Mungbean is predominantly cultivated on soils with residual moisture from previous rainy seasons. Terminal drought stress, especially at the end of the growing season, is a major constraint restricting the yield of mungbean [8, 10 and 93]. The mungbean genotypes with longer primary root and larger total surface area could perform better in terms of water and nutrient acquisition, especially when water and nutrients are heterogeneously distributed across different soil levels. This research identified deeper rooting genotypes, like EC862594, IC 616203, IC 616109, IC616184 and EC 862589 (Supplementary Table 2), that could access water from deeper soil layers whenever the topsoil dries up later during the season.  The root morphology traits vary from species to species and also between different genotypes of a species [65, 94-95]. Root architectural features such as TSA, TRL, ARD, PRL, and TRV were responsible for most of the observed root trait variability at the seedling level. Correlation studies showed a positive correlation among TRL, PRL, TPA, TRV, TSA, TRT and TRF. The stated traits also exhibited a negative correlation with average root diameter (ARD). Selection of higher ARD values would negatively affect the root traits mentioned above. For mitigating abiotic stress, increasing root diameter would be an option [96].. The root surface area and root length are mainly influenced by the root diameter [97].  Water and nutrient uptake capability are determined by root architecture. In early stages, genotypes with vigorous root growth (TRL and TSA) take up water and minerals more effectively and have better seedling establishment [98], resulting in increased photosynthetic ability, higher output of biomass, and higher survival rate under stressful conditions. The root number and TRL [99-100] are positively correlated with yield and biomass.  Our analysis showed that among the diameter classes large parts of the mungbean root system consist of a variety of very fine and fine roots in diameters between 0.5 and 2.0 mm. The absorption of water and nutrients, the involvement of very fine and fine roots is well established [64,101-102]. In the root system, a high percentage of fine roots contributes to an improvement in TSA for the acquisition of more water and nutrients and helps plants to cope with stress [103].  The root to shoot ratio (RSR) is also used to predict distribution of biomass among roots and shoots [104]. In rice seedlings, water deficit situation raises root-to-shoot ratio by altering enzymatic activity and carbohydrate balancing [105]. EC 862602, EC862588, and IC 616169 are genotypes identified with higher RSR values (Table 5 and Supplementary Table 2). In the phosphorus efficiency studies, higher ratios of root to shoots are often labelled as index trait because with the improvement in root biomass and the large deep root system required to extract more nutrients [106-107].  For TSA and TRV, genotypes in clusters V and III are diverse (Fig. 3). The rate of absorption of nutrients is dependent on the TSA and TRV [108-109]. The root traits TSA, TRL, and TRV were the target traits for mungbean to increase the efficiency of nutrient use (especially phosphorus) at the seedling stage [110]. Increasing nitrogen efficiency in maize, the RDW and TRL played an important role [111]. IC 862615, EC 862617, IC616200 and IC616150 were identified with having higher RDW (Table 5, and Supplementary Table 2). In case of finger millet, the starvation reaction to phosphorus is mitigated by increased TRL and root hair count and length [112].  A thorough understanding of the multiple associations between root traits [113] is needed for the proper use of root traits in crop breeding. In order to demonstrate the relationship and diversity of the characteristics and relative homogeneous grouping of genotypes based on root traits, our research examined principal component analysis, hierarchical clustering, Pearson's correlation, and Shanon-Weaver diversity indices. The variations contained in the mungbean mini-core for PRL, TSA, TPA, TRT and TRF have potential for mungbean improvement programmes and genotype categorization in high trait value, medium trait value and low trait value classes based on mean and SD facilitates the selection of breeding materials [114-115].  The Shannon-Weaver diversity index (H') showed phenotypic diversity among the root characteristics, where low H' values stand for unbalanced frequency distribution and lack of trait diversity[116], while high H' means high genetic diversity in traits[117]. In the mungbean mini-core, root characteristics i.e., TRL, TSA, LPV, and TRF had a relatively high level of H '(>0.9) showing high diversity for these characteristics. The high H 'value and the positive correlation of these characteristics with PRL and TPA showed that these characteristics are appropriate at the seedling stage to improve water and nutrient uptake efficiency in mungbean. In addition, variations in ARD, TRV, and TRT would also be useful in stressful environments to increase nutrient productivity and crop yields [118].  The genetic and molecular basis of the root system architecture and its plasticity in drought conditions has been documented in major legumes. QTLs for root surface area [30] and root length[31] in soybean, root diameter in cowpea[34], root length in pea[35], basal root angle in common bean [36], and rooting depth, root surface area and root length density in chickpea [37] and root surface area, lateral root number and specific root length[119] in lentil have been reported. For other essential crops such as Rice [120-121], durum wheat [122], barley [123-124], maize [125], sorghum [126], pearl millet [127], finger millet [112],and cotton [128] very significant progress has been made in the understanding and use of root traits in breeding programmes. The ideal root architectural ideotype for abiotic stress tolerance for optimised nutrient and water acquisition – and even coining the phrase '...steep, cheap and deep...' [129] Steep (root angle) [130], cheap (metabolic costs) [131] and deep (root architectural arrangements)[46, 132] .  Mungbean genotypes identified in this experiment with such a wide variety of root properties could be used for subsequent studies in green houses and on field assessment. Finally, the development of mapping population, use of molecular markers technology, root simulations and gene mapping to develop germplasm with improved root traits for better tolerance to water deficit and harsh conditions. |
| 4 | Also, it will be good if the authors can check/edit the entire MS documents for English language and grammar usage. | As suggested, the English language editing and the use of grammar have been reviewed and integrated by our colleagues (Dr. Roland Schafleitner and  Dr. Ramakrishnan M. Nair from AVRDC) |

**Response to Reviewer 2:**

| **Reviewer#2:** | **Reply** |
| --- | --- |
| Line 40: Per hectare? | **Correction was carried out by addition Per hectare** |
| Line 42: Remove "that result in reduced grain yields" | **Removed** |
| Line 43-51: This is far fetched and more generalized or vague. More evidence needed to show that effects of changes in global temperatures actually impacted Mungbean production. | **Agreed with reviewers view. Sentence is deleted.** |
| Line 48-51: Provide references for each of the factor mentioned here that affects Mung bean production. | **References are provided as per suggestion.**  Mungbean, in comparison to other pulses, is relatively heat and drought tolerant, but in spite of that, production is affected by severe abiotic stress, such as low or high temperatures [10], insufficient or excessive water [11-12], high salinity [10], low soil fertility [13] and polluted heavy metal containing soils [14-15] and ultraviolet-B(UV-B) radiation[16]. |
| Line 53: Remove "Crop breeding programmes mainly have focused on above-ground organs". | **As per suggestions , sentence was removed** |
| Line 56-57: Would expect a brief description on some screening techniques that are being developed and proven useful at least latest. Also a sentence or two is needed to know why earlier methods weren't useful. | **As per suggestions, screening techniques available and their drawbacks were added and entire introduction was rewritten.**    In order to examine root features, several phenotyping techniques were documented, particularly hydroponic systems utilizing growth bags (or germination sheet)[52-54], agar-plate and aeroponic systems [55] , soil rhizotrons [56-58], deep column methods[59], transparent containers[60],PVC pipes (columns) and glass-walled rhizoboxes filled with soil, but these methods are costly, require considerable labour and large area for phenotyping larger genotype sets[61-63]. The unique semi-hydroponic phenotyping system[64] was established to assess the heterogeneity of the root trait in the narrow-leaf lupin (*Lupinus angustifolius* L.) core set[65-66].The same technique was used for root phenotyping in chickpea (*Cicer arietinum* L.)[67], maize (*Zea mays* L.) and barley (*Hordeum vulgare* L.)[68]. This unique semi-hydroponic technique was modified to suit the purpose to screen a mungbean mini-core collection for root traits.  The use of digital imaging and software tools for root image analysis is an innovative and efficient way to accurately assess root traits [69-71]. Several software programmes are available to extract two-dimensional root morphology traits. This varies from the manual root label DART [70], commercially available software and semi-automated root analysis tool WinRhizo^TM^ (Regent Instruments, Québec, Canada) (Pro, 2004) and EzRhizo[72], freely usable, fully integrated and automated SmartRoot [73] software for small root systems. |
| Line 58-61: How does it connect with the preceding statements on Screening techniques, does the author used new techniques to report the findings what he did? | **Yes the sentence is not connecting. The entire introduction is rewritten and in material and methods, modified semi-hydroponic system was described.**  The unique semi-hydroponic phenotyping system[64] was established to assess the heterogeneity of the root trait in the narrow-leaf lupin (*Lupinus angustifolius* L.) core set[65-66].The same technique was used for root phenotyping in chickpea (*Cicer arietinum* L.)[67], maize (*Zea mays* L.) and barley (*Hordeum vulgare* L.)[68]. This unique semi-hydroponic technique was modified to suit the purpose to screen a mungbean mini-core collection for root traits.  **In material and methods**  **A Modified Semi-hydroponic system**  To suit the experimental purpose, the semi-hydroponic technique [64] has been modified. Our fundamental goal was to screen larger germplasm sets for a shorter period of time. This was accomplished by changing the bin size with smaller plastic trays and germination stands, resulting in a 26 cm long germination stand with 12 cm width and 8 cm height (specifically created by Bio-Link Pvt. Ltd. New Delhi). The size of the plastic tray was 51 cm in length, 43 cm in width, and 13 cm in height (Tarson products Pvt. Ltd.). Germination paper (SGPK-145; GSM with the creepy surface was obtained from Bio-Link Pvt. Ltd New Delhi) with a size of 14 x 8 cm was used. Two equally cut germination paper sizes (length 14 cm x height 10 cm) were positioned in each germination cell to accommodate seeds for germination. Eight litres of double distilled water were used to moisten the germination paper stand in the plastic tray. For each accession, 10 seeds were used for germination in each germination cell. In each germination stand, 12 germplasm accessions were housed (Fig.1). Three germination stands were placed in each plastic tray, to grow thirty-six mungbean accessions. A total of 9 plastic trays, each containing 36 accessions, covered 296 mini-core collection entries. Due to hard seeds produced during seed multiplication in the field, we were at risk of poor germination. In order to prevent variable germination rate, uniform 10 seeds were used for germination [75]. Three uniform, healthy seedlings were selected from the emerging seedlings as three biological replicates after 20 days. |
| Line 62: A brief description of ideal root architecture attributes is needed. | **As per suggestions a brief description of ideal root architecture attributes were described.**  **Under introduction:**  The ideal root phenotypes under water deficit situations, in food grain legumes include root surface area [30], root length [31], deeper and proliferative roots [32-33] in soybean (*Glycine max* L.), root diameter in cowpea (*Vigna unguiculata* L.) [34], root length in pea (*Pisum sativum* L.) [35], basal root angle in common bean (*Phaseolus vulgaris* L.) [36], and rooting depth, root surface area, root length density [37] and proliferative & deeper roots [38-39] in chickpea (*Cicer arietinum* L.). Other crops which have benefited from ideal root phenotypes like proliferative and deep root traits under drought tolerance include rice (*Oryza sativa*), [40-41], maize (*Zea mays*) [42-43], barley (*Hordeum vulgare*) [44] and wheat (*Tritcum aestivum)* [45-46].  **Discussion:** The ideal root architectural ideotype for abiotic stress tolerance for optimised nutrient and water acquisition and even coining the phrase '...steep, cheap and deep...' [129] Steep (root angle) [130], cheap (metabolic costs) [131] and deep (root architectural arrangements) [46, 132]. |
| Line 76-78: Introduction is plagued with more generalised statements and doesn't discuss the topic in depth, the topic is about Mungbean but not a sentence shows what issues are with Mungbean root adaptation and why one should explore a global germplasm to find variation for roots. The next important theme of this study is root phenotyping platforms, there are many statements about this but not exclusive or conclusive explanation provided, therefore the introduction may need restructuring inclusive of these topics to ensure flow with the references. | **Introduction is rewritten by incorporating all suggestions.**  Mungbean is cultivated on 7 million hectares worldwide [1]. Among six Asiatic *Vigna* species, green gram or mungbean (*Vigna radiata*) is the most widely distributed species [2]. It is a major food grain legume and cash crop widely cultivated in South, East and South East Asia and is increasingly grown in South America and sub-Saharan Africa.. It fits in many intense cropping systems due to its photo-insensitivity and short duration. Mungbean is rich in easily digestible proteins, carbohydrates, fibres, minerals, vitamins, antioxidants and other phytonutrients [3-5], giving the crop potential to be used for mitigating malnutrition [6]. Mungbean is consumed as grain e.g. in dhal, as a sprout or in countless other preparations, as whole or split grains. The yield potential of the mungbean is about 2 tonnes per hectare, while the average productivity is 0.5 tonnes per hectare. The yield gap is due to cultivation on marginal land, suboptimal crop management, and abiotic and biotic stresses [7-9].  Mungbean, in comparison to other pulses, is relatively heat and drought tolerant, but in spite of that, production is affected by severe abiotic stress, such as low or high temperatures [10], insufficient or excessive water [11-12], high salinity [10], low soil fertility [13] and polluted heavy metal containing soils[14-15] and ultraviolet-B(UV-B) radiation[16].  Mungbean is mainly grown in three seasons on the Asian continent, spring (February / March) summer (March / April) and Kharif (June / July). Erratic water supply during these months exposes the seedlings to water stress when grown in rainfed conditions. Scarcity of water imposes stress at any plant stage [17]. Yields in tropical and subtropical countries such as India, Pakistan and Ethiopia, will decline due to an expected higher incidence of water deficits [18]. The expansion of the global drought-restrained zone is threatening crop production [19]. Insufficient availability of water on its own is more critical than any other environmental trigger for the development of mungbean [20]. Water scarcity during the seedling stage hinders the establishment of healthy seedlings and limits yield. Drought produces many devastating effects on plants by disrupting various plant activities, such as carbon assimilation, reduced turgor, enhanced oxidative damage, and modifications in leaf gas exchange, resulting in a reduction in yield [21].Better water supply for plants is critical for boosting crop production in spite of water shortages [22].  Root system architecture is seen as the main factor for efficient water absorption and therefore for maintaining productivity in conditions of drought [23]. Root features are commonly known as the root system architecture (RSA), which refers to the form of the roots and their physical space. With its ability to obtain more water from the soil, a deeper and more proliferative RSA is also capable of avoiding water deficit conditions.  Roots begin to spread into deeper soil layers as plants encounter water deficit stress [24-25].The root diameter and distribution of root conductivity-regulating metaxyl vessels are also documented to provide drought tolerance in food grain legumes [26]. Thicker roots prefer to penetrate deeper into soil layers [27].  The key determinants of proliferative rooting are the initiation and elongation of the lateral root, which usually refers to the sum of the lateral root, root volume, root surface and root length density. Proliferative and deeper roots have increased capacity for water absorption in water-deficient soils [28-29]. The ideal root phenotypes under water deficit situations, in food grain legumes include root surface area [30], root length [31], deeper and proliferative roots [32-33] in soybean (*Glycine max* L.), root diameter in cowpea (*Vigna unguiculata* L.) [34], root length in pea (*Pisum sativum* L.) [35], basal root angle in common bean (*Phaseolus vulgaris* L.) [36], and rooting depth, root surface area, root length density [37] and proliferative & deeper roots [38-39] in chickpea (*Cicer arietinum* L.). Other crops which have benefited from ideal root phenotypes like proliferative and deep root traits under drought tolerance include rice (*Oryza sativa*), [40-41], maize (*Zea mays*) [42-43], barley (*Hordeum vulgare*) [44] and wheat (*Tritcum aestivum)* [45-46] .  Changing the root system architecture can improve desirable agronomic attributes such as yield, drought tolerance and tolerance to nutrient deficiencies [47-49] and incorrect phenotyping and small mapping population sizes hinder the use of genomics to improve root characteristics in breeding programmes [50].In order to translate current physiological and genetic breakthroughs into improvements of yield and productivity especially in dry ecosystems, precise phenotyping and evaluation of root-related traits is vitally important. An effective approach for increasing adaptation to edaphic stress is the selection and breeding cultivars with root systems that more effectively use nutrients and water than current varieties [51]  In order to examine root features, several phenotyping techniques were documented, particularly hydroponic systems utilizing growth bags (or germination sheet)[52-54], agar-plate and aeroponic systems [55] , soil rhizotrons [56-58], deep column methods[59], transparent containers[60],PVC pipes (columns) and glass-walled rhizoboxes filled with soil, but these methods are costly, require considerable labour and large area for phenotyping larger genotype sets[61-63] . The unique semi-hydroponic phenotyping system[64] was established to assess the heterogeneity of the root trait in the narrow-leaf lupin (*Lupinus angustifolius* L.) core set[65-66].The same technique was used for root phenotyping in chickpea (*Cicer arietinum* L.)[67], maize (*Zea mays* L.) and barley (*Hordeum vulgare* L.)[68]. This unique semi-hydroponic technique was modified to suit the purpose to screen a mungbean mini-core collection for root traits.  The use of digital imaging and software tools for root image analysis is an innovative and efficient way to accurately assess root traits [69-71]. Several software programmes are available to extract two-dimensional root morphology traits. This varies from the manual root label DART [70], commercially available software and semi-automated root analysis tool WinRhizo^TM^ (Regent Instruments, Québec, Canada) (Pro, 2004) and EzRhizo[72], freely usable, fully integrated and automated SmartRoot [73] software for small root systems.  The World Vegetable Center (WorldVeg) has established a mungbean mini-core set, which represents a large proportion of the diversity available for this species in the WorldVeg gene bank [74] This resource comprises a major genetic resource for identifying new traits for future use in breeding programmes. Characterising the genotypic variability of the biodiverse accessions of the mungbean mini-core set for variation of root characteristics is the first step for identifying root traits for use in breeding more water and nutrient-use efficient varieties. The present study determined the genotypic variation of root characteristics in the mungbean mini-core set using a modified semi-hydroponic system and resulted in grouping of the germplasm based on key root traits. |
| Line 84: In field or glasshouse? | In field |
| Line 85: Is it Conviron growth chamber, if so or not please provide details of the growth chamber. | The Conviron growth chamber and Model PGW 36 with growth area 3.3 m2 (36 ft^2^) Made In Canada. |
| Line 86: move the abbreviation to line 84 | Moved abbreviation to line 84 |
| Line 95: Please add from where you purchased these items, germination stand, trays, germination paper. | Germination stand was custom prepared as per specifications by Bio-Link pvt Ltd. New Delhi. The germination paper was of type SGPK-145; GSM with creepy surface was procured from Bio-Link Pvt. Ltd New Delhi. |
| Line 98: Cannot resolve how you managed three replications here, 9*3*12=324, Total number of accessions are 296 (line 83) and 3 replications of each would make = 888 accessions. Please clarify. | **Below is the clarification**  For each accession, 10 seeds were used for germination in each germination cell. In each germination stand, 12 germplasm accessions were housed (Fig.1). Three germination stands were placed in each plastic tray, to grow thirty-six mungbean accessions. A total of 9 plastic trays, each containing 36 accessions, covered 296 mini-core collection entries. |
| Line 111: Any version number or manufacturer details. | Epson V700 |
| Line 115: Replace the title with Assessment of root phenotypes. | Replaced:  Assessment of **root phenotypes** in mungbean mini-core collection (MMC) from World Vegetable Center (AVRDC) Taiwan |
| Line 126: please provide instrument details | **Details: Digital weighing balance from Citizen company and model number is CX 265.** |
| Line 145: Material and methods lacks bit of clarity on how the experiments were conducted particularly information on replications is ambiguous. Further lacks the description of the process of how the measurements were undertaken. There are different equipment used in this study but none of them have manufacture details. | **As per suggestions the details of instrument were added and clarity on replication were explained:**  **Material and Methods:**  Plant material for this analysis included the WorldVeg mini-core collection of 296 genotypes [74] collected from 18 countries around the globe. The seeds were procured from the National Plant Genetic Resources Bureau (NBPGR), New Delhi (Table S1). The obtained seed was multiplied in the field during the 2018 rainy season at the Indian Agricultural Research Institute (IARI) in New Delhi (Latitude 28° 38' 31.9236" N and Longitude 77° 9' 16.434"). The climate-controlled growth chamber (CONVIRON, Canada, PGW 36 with a growth area of 3.3 m^2^ (36 ft^2^)) at the National Phytotron Facility (NPF) in IARI, New Delhi, India was used for experiments. The studies were performed between September 2019 and December 2019. The day/night temperature of 30/18^o^C, 12 h photoperiod, and 90 % relative humidity were maintained in the growth chamber. Seeds were surface-sterilized for 3 minutes in 0.1 % HgCl_2_ and then rinsed in double-distilled water before being kept for germination in a modified semi-hydroponics system.  **Modified Semi-hydroponic system**  To suit the experimental purpose, the semi-hydroponic technique [64] has been modified. Our fundamental goal was to screen larger germplasm sets for a shorter period of time. This was accomplished by changing the bin size with smaller plastic trays and germination stands, resulting in a 26 cm long germination stand with 12 cm width and 8 cm height (specifically created by Bio-Link Pvt. Ltd. New Delhi). The size of the plastic tray was 51 cm in length, 43 cm in width, and 13 cm in height (Tarson products Pvt. Ltd.). Germination paper (SGPK-145; GSM with the creepy surface was obtained from Bio-Link Pvt. Ltd New Delhi) with a size of 14 x 8 cm was used. Two equally cut germination paper sizes (length 14 cm x height 10 cm) were positioned in each germination cell to accommodate seeds for germination. Eight litres of double distilled water were used to moisten the germination paper stand in the plastic tray. For each accession, 10 seeds were used for germination in each germination cell. In each germination stand, 12 germplasm accessions were housed (Fig.1). Three germination stands were placed in each plastic tray, to grow thirty-six mungbean accessions. A total of 9 plastic trays, each containing 36 accessions, covered 296 mini-core collection entries. Due to hard seeds produced during seed multiplication in the field, we were at risk of poor germination. In order to prevent variable germination rate, uniform 10 seeds were used for germination [75]. Three uniform, healthy seedlings were selected from the emerging seedlings as three biological replicates after 20 days.  Each tray was filled with 8 litres of modified Hoagland solution when cotyledon leaves were developed. The basic nutrient solution consisted of 0.92 mM K_2_SO_4_, 1 mM MgSO_4_, 5 mM urea, 0.75 mM CaCl_2_.2H_2_O, 0.04 mM Fe-EDTA) and micronutrients (0.62 μM CuSO_4_), 0.6 μM ZnSO_4_, 2.4 μM H_3_BO_3_, 0.6 μM Na_2_MoO_4_ and 0.9 μM MnSO_4_ [76]. The pH of the nutrient solution was maintained at 6.0 with 1 M HCl or 1 M KOH for adjustment. The solution in the trays was replaced on alternating days and the entire system was periodically aerated by small aquarium air pumps (SOBOTM TM, 5W, 2-way air pump with 2 nozzles, 4.2 W and 2 x 5.5 L, output power).  **Root scanning for image capture**  To capture root images, twenty days old seedlings were used. The intact root system was harvested from each plant and carefully spread, without overlapping roots, over a scanning tray of a root scanner (EPSON^TM^ V700). TIFF-format grayscale quality images were analysed by WinRhizo^TM^ (Pro version 2016; Regent Instrument Inc., Quebec, Canada). Setup parameters: image resolution of 400 dpi, manual dark root on white background, scanner calibration, 8 bit depth, image resolution of 4395 x 6125 pixels, 0 mm focal length. Roots are distributed in a 30 x 40 x 2 cm acrylic tray with a volume of 700 mlwater. Manually, the debris were separated from the sample roots by suspension in a beaker containing water. The trash-free clean roots were used for scanning.  **Root image analysis:**  Total root length (TRL), total surface area (TSA), primary root length (PRL), total root volume (TRV), root average diameter (RAD), total root tips (TRT), total root forks (TRF), and total root crossings (TRC)were the major root traits analysed by the WinRhizo^TM^ software . PRL was measured manually by means of a steel measurement scale (EISCO-GROZTM).  The WinRhizo^TM^ provided main and additional data to classify total root length (TRL), total root volume (TRV),total root surface area (TSA), and total root tips (TRT) into five classes of root diameter intervals: class1: (0-0.5 mm), class 2: (0.5-1.0 mm), class 3: (1.0-1.5 mm), class 4: (1.5-2.0 mm) and class 5: (>2.0 mm)[77-79]. In every class, the root traits were calculated as a proportion of the total trait [68]. The details of each tested trait are given in Table 1. Biomass related traits like root dry weight (RDW) (mg), shoot dry weight (SDW) (mg), and total dry weight (TDW) (mg) were determined using a digital weighing balance (Citizen^TM^,CX 265) on three biological replicates after air-forced drying in an oven at 70 °C for 72 h. |
| Line 201-206: Topic for Discussion section | **As per suggestion this was moved to discussion section :**  Genotypes of the diverse clusters II, VI, and VIII are candidates for crossing programmes to generate good recombinants for root traits like PRL, TSA, TRL, TPA, and TRT. Based on the cluster analysis the cross combinations (EC 862594 (C-II) x IC 616154(C-VI), TSA (IC 616154(C-II) x EC 862622(C-VI), TRL (EC 862670(C-VII) x EC 862662(C-VI), TPA(IC 15252(C-VII) x IC 616154(C-VI)) will be useful to improve PRL. |
| Line 208-215: This paragraph is not necessary; you may start with your aim and focus on the main outcomes of the study. | **As per suggestions paragraph is restructured to start aim and focus on outcome of the study**  Across both breeding programmes and scientific research, the quest for root characteristics has intensified, offering improvement in the acquisition of resources and tolerance to abiotic stress including heat and drought stress, especially in resource-poor environments. Advancement was delayed due to problems in effective and accurate root trait phenotyping in large germplasm panels [49-50, 87]. In a mini-core set of 296 mung bean genotypes, the use of a modified semi-hydroponic phenotyping system saved time and space for phenotyping and provided access to substantial variation of root traits.  The novel method of semi-hydroponic phenotyping [64] has been modified to screen and explore the genetic variation of different root characters at the early vegetative stage of a mungbean mini-core set (Table 2, S1 Table). Previously, the semi-hydroponic phenotyping platform provided quality data for the wild narrow leaf lupin[65], core collections of lupin[66], and chickpea [67] to assess the genetic basis of variation in root characteristics. |
| Line 226: Although few references are listed, the section does not provide or discuss the disadvantages of previous methods and a comparison with proper examples for both methods needed to make it more convincing. | This part is rewritten to provide the disadvantages of previous methods and a comparison with proper examples for both methods in introduction and discussion.  **In Introduction:**  In order to examine root features, several phenotyping techniques were documented, particularly hydroponic systems utilizing growth bags (or germination sheet)[52-54], agar-plate and aeroponic systems [55] , soil rhizotrons [56-58], deep column methods[59], transparent containers[60],PVC pipes (columns) and glass-walled rhizoboxes filled with soil, **but these methods are costly, require considerable labour and large area for phenotyping larger genotype sets[61-63] .** The unique semi-hydroponic phenotyping system[64] was established to assess the heterogeneity of the root trait in the narrow-leaf lupin (*Lupinus angustifolius* L.) core set[65-66].The same technique was used for root phenotyping in chickpea (*Cicer arietinum* L.)[67], maize (*Zea mays* L.) and barley (*Hordeum vulgare* L.)[68]. This unique semi-hydroponic technique was modified to suit the purpose to screen a mungbean mini-core collection for root traits.  The use of digital imaging and software tools for root image analysis is an innovative and efficient way to accurately assess root traits [69-71]. Several software programmes are available to extract two-dimensional root morphology traits. This varies from the manual root label DART [70], commercially available software and semi-automated root analysis tool WinRhizo^TM^ (Regent Instruments, Québec, Canada) (Pro, 2004) and EzRhizo[72], freely usable, fully integrated and automated SmartRoot [73] software for small root systems.  **Discussion:** The novel method of semi-hydroponic phenotyping [64] has been modified to screen and explore the genetic variation of different root characters at the early vegetative stage of a mungbean mini-core set (Table 2, Supplementary Table 1). **Previously, the semi-hydroponic phenotyping platform provided quality data for the** wild narrow leaf lupin[65], core collections of lupin[66], and chickpea [67] to assess the genetic basis of variation in root characteristics. |
| Line 228: Any reference? | References were added:  **The root morphology traits vary from species to species and also between different genotypes of a species [65, 93-94].**  Chen YL, Dunbabin VM, Diggle AJ, Siddique KH, Rengel Z. Assessing variability in root traits of wild *Lupinus angustifolius* germplasm: basis for modelling root system structure. Plant and Soil. 2012 May 1;354(1-2):141-55.  Clements JC, White PF, Buirchell BJ. The root morphology of *Lupinus angustifolius* in relation to other *Lupinus* species. Australian Journal of Agricultural Research. 1993;44(6):1367-75.  Brück H, Sattelmacher B, Payne WA. Varietal differences in shoot and rooting parameters of pearl millet on sandy soils in Niger. Plant and soil. 2003 Apr 1;251(1):175-85. |
| Line 232: What does this mean, helpful or not useful for benefit of mungbeans? | **In case of mungbean, selection for higher ARD will decrease other root traits due to negative correlation. Smaller the root diameter will be more useful for plants.**  **In discussion:**  The stated traits also exhibited a negative correlation with average root diameter (ARD). Selection of higher ARD values would negatively affect the root traits mentioned above. Plants with a smaller root diameter and a specific root length of fine roots are found to be better suited to dry conditions [95]. The root surface area and root length are mainly influenced by the root diameter [96]. |
| Line 242: examples from any pulse crop may further strengthen your statements. | **As per suggestion references from pulse crops were added to support statements.**  The genetic and molecular basis of the root system architecture and its plasticity in drought conditions has been documented in major legumes. QTLs for root surface area [30] and root length[31] in soybean, root diameter in cowpea[34], root length in pea[35], basal root angle in common bean [36], and rooting depth, root surface area and root length density in chickpea [37] and root surface area, lateral root number and specific root length[118] in lentil have been reported. |
| Line 246: Again this is another generalized statement, please briefly state what it means as many readers would be interested. | **As per suggestions sentences were restructured to convey proper meaning**  Our analysis showed that among the diameter classes large parts of the mungbean root system consist of a variety of very fine and fine roots in diameters between 0.5 and 2.0 mm. The absorption of water and nutrients, the involvement of very fine and fine roots is well established [64,100-101]. In the root system, a high percentage of fine roots contributes to an improvement in TSA for the acquisition of more water and nutrients and helps plants to cope with stress [102]. |
| Line 277: Discussion needs bit more on mungbean literature about which root traits were previously researched and whether selection for those traits proved beneficial or failure. Report some examples of existing varieties that are susceptible to abiotic stress and where root parameters were considered as a reason. This information may provide to convince readers why you may need to explore root variation in a global germplasm. I'm convinced that this study is not just to test a new screening method in mungbeans. Although there are some good statistical classifications evaluated to divide the variation into clusters, it would be good to provide a list of lines with ideal root phenotypes and also provide a comment on undesirable phenotypes. | **Discussion was rewritten to address the concerns.**  **Table 5 was included as per suggestion for having top five lines with ideal root phenotypes and bottom five with undesirable root phenotypes.**  **Discussion :**  Across both breeding programmes and scientific research, the quest for root characteristics has intensified, offering improvement in the acquisition of resources and tolerance to abiotic stress including heat and drought stress, especially in resource-poor environments. Advancement was delayed due to problems in effective and accurate root trait phenotyping in large germplasm panels [49-50, 87]. In a mini-core set of 296 mung bean genotypes, the use of a modified semi-hydroponic phenotyping system saved time and space for phenotyping and provided access to substantial variation of root traits.  The novel method of semi-hydroponic phenotyping [64] has been modified to screen and explore the genetic variation of different root characters at the early vegetative stage of a mungbean mini-core set (Table 2, Supplementary Table 1).Previously, the semi-hydroponic phenotyping platform provided quality data for the wild narrow leaf lupin[65], core collections of lupin[66], and chickpea [67] to assess the genetic basis of variation in root characteristics.  The mungbean root system is similar those of other dicotyledonous species including Arabidopsis, Medicago, and other legumes like chickpea and develops through consecutive branch/lateral root orders from a primary root that emerges from the embryo[88]. The volume and size of lateral branches/roots is an important contributor for the growth and development of food grain legumes [89].  Depth of rooting and density of root branches are essential architectural features that directly affect water and nutrients acquisition in the soil strata [90]. The PRL of the deep-rooting genotype EC862594 was more than twice as high than the average value of the whole germplasm set and genotype EC 862670 had a more than 3-fold TRL than the average, corroborating the trait diversity present in the mini-core collection (Supplementary Table 2). Another aspect that affects deep rooting is root penetrability and root thickness or root diameter [91]. The thicker roots prefer to go deeper into the soil in order to obtain water from deeper soil layers [27].Research shows that in chickpea there is a strong association between prolific root system and the development of grain in terminal drought situations [38].In the case of total surface area, the best genotype IC 616276 only had about 20% more TSA than the average. This suggests that the TSA exhibited lower variations in the Mungbean mini-core collection. (Table 5 and Supplementary Table 2).  Proliferative rooting is primarily characterised by the initiation and elongation of lateral roots, which usually refers to the number of the lateral root, the root length density (RLD), and the root volume and the root surface area. Proliferative roots have a considerably large water absorption potential in water deficit soils. In environments with water scarcity, lines with higher RLD showed clearly improved yield and drought-tolerance-related performance [92].  Mungbean is predominantly cultivated on soils with residual moisture from previous rainy seasons. Terminal drought stress, especially at the end of the growing season, is a major constraint restricting the yield of mungbean [8, 10 and 93]. The mungbean genotypes with longer primary root and larger total surface area could perform better in terms of water and nutrient acquisition, especially when water and nutrients are heterogeneously distributed across different soil levels. This research identified deeper rooting genotypes, like EC862594, IC 616203, IC 616109, IC616184 and EC 862589 (Supplementary Table 2), that could access water from deeper soil layers whenever the topsoil dries up later during the season.  The root morphology traits vary from species to species and also between different genotypes of a species [65, 93-94]. Root architectural features such as TSA, TRL, ARD, PRL, and TRV were responsible for most of the observed root trait variability at the seedling level. Correlation studies showed a positive correlation among TRL, PRL, TPA, TRV, TSA, TRT and TRF. The stated traits also exhibited a negative correlation with average root diameter (ARD). Selection of higher ARD values would negatively affect the root traits mentioned above. Plants with a smaller root diameter and a specific root length of fine roots are found to be better suited to dry conditions [95]. The root surface area and root length are mainly influenced by the root diameter [96].  Water and nutrient uptake capability are determined by root architecture. In early stages, genotypes with vigorous root growth (TRL and TSA) take up water and minerals more effectively and have better seedling establishment [97], resulting in increased photosynthetic ability, higher output of biomass, and higher survival rate under stressful conditions. The root number and TRL [98-99] are positively correlated with yield and biomass.  Our analysis showed that among the diameter classes large parts of the mungbean root system consist of a variety of very fine and fine roots in diameters between 0.5 and 2.0 mm. The absorption of water and nutrients, the involvement of very fine and fine roots is well established [64,100-101]. In the root system, a high percentage of fine roots contributes to an improvement in TSA for the acquisition of more water and nutrients and helps plants to cope with stress [102].  The root to shoot ratio (RSR) is also used to predict distribution of biomass among roots and shoots [103]. In rice seedlings, water deficit situation raises root-to-shoot ratio by altering enzymatic activity and carbohydrate balancing [104]. EC 862602, EC862588, and IC 616169 are genotypes identified with higher RSR values (Table 5 and Supplementary Table 2). In the phosphorus efficiency studies, higher ratios of root to shoots are often labelled as index trait because with the improvement in root biomass and the large deep root system required to extract more nutrients [105-106].  For TSA and TRV, genotypes in clusters V and III are diverse (Fig. 3). The rate of absorption of nutrients is dependent on the TSA and TRV [107-108]. The root traits TSA, TRL, and TRV were the target traits for mungbean to increase the efficiency of nutrient use (especially phosphorus) at the seedling stage [109]. Increasing nitrogen efficiency in maize, the RDW and TRL played an important role [110]. IC 862615, EC 862617, IC616200 and IC616150 were identified with having higher RDW (Table 5, and Supplementary Table 2). In case of finger millet, the starvation reaction to phosphorus is mitigated by increased TRL and root hair count and length [111].  A thorough understanding of the multiple associations between root traits [112] is needed for the proper use of root traits in crop breeding. In order to demonstrate the relationship and diversity of the characteristics and relative homogeneous grouping of genotypes based on root traits, our research examined principal component analysis, hierarchical clustering, Pearson's correlation, and Shanon-Weaver diversity indices. The variations contained in the mungbean mini-core for PRL, TSA, TPA, TRT and TRF have potential for mungbean improvement programmes and genotype categorization in high trait value, medium trait value and low trait value classes based on mean and SD facilitates the selection of breeding materials [113-114].  The Shannon-Weaver diversity index (H') showed phenotypic diversity among the root characteristics, where low H' values stand for unbalanced frequency distribution and lack of trait diversity[115], while high H' means high genetic diversity in traits[116]. In the mungbean mini-core, root characteristics i.e., TRL, TSA, LPV, and TRF had a relatively high level of H '(>0.9) showing high diversity for these characteristics. The high H 'value and the positive correlation of these characteristics with PRL and TPA showed that these characteristics are appropriate at the seedling stage to improve water and nutrient uptake efficiency in mungbean. In addition, variations in ARD, TRV, and TRT would also be useful in stressful environments to increase nutrient productivity and crop yields [117].  The genetic and molecular basis of the root system architecture and its plasticity in drought conditions has been documented in major legumes. QTLs for root surface area [30] and root length[31] in soybean, root diameter in cowpea[34], root length in pea[35], basal root angle in common bean [36], and rooting depth, root surface area and root length density in chickpea [37] and root surface area, lateral root number and specific root length[118] in lentil have been reported. For other essential crops such as Rice [119-120], durum wheat [121], barley [122-123], maize [124], sorghum [125], pearl millet [126], finger millet [111], and cotton [127] very significant progress has been made in the understanding and use of root traits in breeding programmes. The ideal root architectural ideotype for abiotic stress tolerance for optimised nutrient and water acquisition and even coining the phrase '...steep, cheap and deep...' [128] Steep (root angle) [129], cheap (metabolic costs) [130] and deep (root architectural arrangements) [46, 131].  Mungbean genotypes identified in this experiment with such a wide variety of root properties could be used for subsequent studies in green houses and on field assessment. Finally, the development of mapping population, use of molecular markers technology, root simulations and gene mapping to develop germplasm with improved root traits for better tolerance to water deficit and harsh conditions. |
| Line 324: Make it italics and follow same all through | **Italics were done and same is followed all through the manuscript.** |
| Line 334: Be consistent, either use abb. or full forms or follow journal guidelines | **Abbreviations were removed and full forms are followed.** |
| Line 562: why only 3 plants tested when 10 plants were actually used. | **Ten seedlings were grown in each accession.**  Among 10 seedlings, 3 were used as biological replicates for screening roots. The reason for planting 10 seedlings are   1. Hard seed is problem in mungbean will lead to poor germination. (Paul D et. al. 2018) 2. Hard seed won’t imbibe water and won’t germinate. 3. Poor germination in diverse panel of mungbean mini-core collection   Paul D, Chakrabarty SK, Dikshit HK, Singh Y. Variation for hardseededness and related seed physical parameters in mung bean (*Vigna radiata* (L.) Wilczek). Indian J. Genet. 2018 Aug 1;78(3):333-41. |
