# Supplementary material for: Assessment of root phenotypes in mungbean mini-core collection (MMC) from the World Vegetable Center (AVRDC) Taiwan
Source: PLoS One. 2021 Mar 4;16(3):e0247810. doi: 10.1371/journal.pone.0247810 (PMC7932546; doi:10.1371/journal.pone.0247810)
Supplement: Attachment — Submitted filename: Response to Reviewers.docx [file pone.0247810.s004.docx]

|  | **Few more minor comments** | **Reply** |
| --- | --- | --- |
| 1 | Line 150 : Strongly recommend inclusion of technical replicates (at least the best performing accessions) for such studies to enhance statistical significance | Technical replicates was included instead of biological replicates |
| 2 | Line 221 : TDW instead of TWD?? | Yes, it is TDW. |
| 3 | Line 222 : Could this line be modified? At the moment, this doesn't align well with the next statement. | Yes, line was modified and rewritten as  While RDW showed positive association with other biomass traits like SDW and TDW. |
| 4 | Line 379 : Consider adding a sentence on a successful on - field establishment of derivatives from some of the previous studies using semi-hydroponic method if available. | There is ample evidence that the entries chosen on the basis of semi-hydroponics or hydroponics are also successful in soil and field experiments.[132-135]  Chen YL, Dunbabin VM, Postma JA, Diggle AJ, Palta JA, Lynch JP, Siddique KH, Rengel Z. Phenotypic variability and modelling of root structure of wild Lupinus angustifolius genotypes. Plant and Soil. 2011 Nov;348(1):345-64.  Baier AC, Somers DJ, Gusiafson JP. Aluminium tolerance in wheat: correlating hydroponic evaluations with field and soil performances. Plant Breeding. 1995 Aug;114(4):291-6.  Gahoonia TS, Nielsen NE. Barley genotypes with long root hairs sustain high grain yields in low-P field. Plant and Soil. 2004 May;262(1):55-62.  Tavakkoli E, Fatehi F, Rengasamy P, McDonald GK. A comparison of hydroponic and soil-based screening methods to identify salt tolerance in the field in barley. Journal of experimental botany. 2012 Jun 13;63(10):3853-67. |
| 5 | Table 2 : Add collection Also add AVRDC before Mungbean mini core collection throughout the manuscript. | Corrected throughout the manuscript. |
| 6 | Table 3 : Repeat above | Yes repeated as suggested |
| 7 | Table 5 : Semi-hydroponic conditions | Yes corrected as suggested |
| 8 | Figure 1: Germination stand?  Also it would be ideal to add a picture of plastic tray mentioned in M & M, the order can be growth chamber (a), plastic tray (b), germination stand (c), and the rest follow in the order they are. | Corrected as per sequence suggested  order can be (a) growth chamber, (b) plastic tray, (c) germination stand |
| 9 | Figure 2 : add "of" in between distribution and root traits. It should be number of accessions on the y- axis rather than frequency | Edited and corrected as per suggestions |
| 10 | Figure 6 : Will recommend to add cluster names as colour legends for this dendrogram, can be made in MS powerpoint | Edited and corrected as per suggestions |
| 11 | Title for S1 Table : Passport data of AVRDC Mungbean mini core collection | Edited and corrected as per suggestions |
| 12 | Title for S2 Table : Mean scores of AVRDC Mungbean mini core collection for fourteen root traits | Edited and corrected as per suggestions |
